# Using survey experiment pretesting to support future pandemic response

**DOI:** 10.1093/pnasnexus/pgae469

**Published:** 2024-10-17

**Authors:** Ben M Tappin, Luke B Hewitt

**Affiliations:** London School of Economics, Houghton St, London WC2A 2AE, United Kingdom; Department of Sociology, Stanford University, 450 Jane Stanford Way, Stanford, CA 94305, USA

**Keywords:** public health communication, survey experiment, cost-effectiveness analysis, COVID-19, pandemic response

## Abstract

The world could witness another pandemic on the scale of COVID-19 in the future, prompting calls for research into how social and behavioral science can better contribute to pandemic response, especially regarding public engagement and communication. Here, we conduct a cost-effectiveness analysis of a familiar tool from social and behavioral science that could potentially increase the impact of public communication: survey experiments. Specifically, we analyze whether a public health campaign that pays for a survey experiment to pretest and choose between different messages for its public outreach has greater impact in expectation than an otherwise-identical campaign that does not. The main results of our analysis are 3-fold. First, we show that the benefit of such pretesting depends heavily on the values of several key parameters. Second, via simulations and an evidence review, we find that a campaign that allocates some of its budget to pretesting could plausibly increase its expected impact; that is, we estimate that pretesting is cost-effective. Third, we find pretesting has potentially powerful returns to scale; for well-resourced campaigns, we estimate pretesting is robustly cost-effective, a finding that emphasizes the benefit of public health campaigns sharing resources and findings. Our results suggest survey experiment pretesting could cost-effectively increase the impact of public health campaigns in a pandemic, have implications for practice, and establish a research agenda to advance knowledge in this space.

**Significance Statement**

The COVID-19 pandemic is estimated to have caused >26 million excess deaths worldwide and the risk of another similar (or worse) pandemic in the near-future is alarmingly high. Here, we estimate that public health campaigns aiming to encourage, for example, vaccination during a pandemic, could cost-effectively increase their impact by using a survey experiment to pretest and choose between different messages for their public outreach. These results have implications for practice and establish a wider research agenda to boost pandemic preparedness.

## Introduction

The COVID-19 pandemic is estimated to have caused an excess 26 million deaths worldwide as of the end of 2023 (1). According to various sources, it is distinctly possible that the world will witness another pandemic with similar or greater capacity for harm in the coming decades (2–6). For example, the UK Government's 2023 National Risk Register estimates a 5–25% likelihood of another reasonable worst-case pandemic within the next 5 years (6). Assessments such as this have motivated a renewed focus on what societies can do to prepare for and respond to future public health emergencies (5, 7–14), including advances in social and behavioral science. To that end, the World Health Organization recently identified public engagement and communication a key area for further research and development to improve future pandemic response (5) (see also, Nuzzo and Ledesma (15)).

In this paper, we respond to these calls by investigating whether public health campaigns could use *survey experiments* to increase the impact of their public outreach; for example, more effectively encouraging vaccination. A survey experiment involves recruiting people online to take a survey, randomizing them into one of the several treatment conditions or a control condition, and then measuring their attitudes, beliefs, and/or behavioral intentions. While survey experiments are of course not a new method in social and behavioral science (16–21), whether and to what extent they could increase the impact of public health campaigns remains unclear, for two interrelated reasons.

First, survey experiments in social and behavioral science are typically used to test hypotheses with the goal of advancing theory. For example, academics may test whether messages in narrative format are more effective than non-narrative messages at changing attitudes or behavior (22, 23). Theories refined by the accumulation of such studies over time can inform public health message development in moments of crisis, as happened during the COVID-19 pandemic (24). However, survey experiments can also be used by public health communicators themselves for another goal: to pretest and choose between several different messages developed for their specific context (25). This message pretesting could be beneficial because evidence suggests that which messages “work best” varies across contexts, sometimes dramatically, and can be difficult to predict from theory or expert advice alone (23, 26–32). Thus, survey experiment pretesting could complement theory-based approaches by enabling practitioners to (i) develop several different candidate messages (based on, e.g. different theories), (ii) quickly identify which one is likely to be most effective in their particular context, and then (iii) choose that message for deploying in their public outreach.

Second, while the practice of using survey experiments to pretest interventions is not a new insight per se, its potential benefit (if any) is unclear, because it depends crucially on assumptions that have not been systematically evaluated. Indeed, this may help explain skepticism toward the method (33). For example, it is not free to run a survey experiment pretest; rather, a public health campaign must pay money to produce multiple messages (e.g. videos for social media) and for the survey experiment to test them. This cost eats into their budget for eventually disseminating their chosen message to the public, thereby diminishing the number of people it can reach. In the limit, if they spend their entire budget on the pretest, it does not matter whether it helps them find a more effective message, because they can no longer afford to disseminate that message to the public and the impact of their campaign is zero by definition. Thus, the budget of the campaign and the cost of pretesting are key assumptions governing the potential benefit of survey experiment pretesting.

Another crucial assumption regards the improvement in message effectiveness campaigns can expect from the pretesting. For example, suppose a campaign develops and pretests five different messages, and selects the one that performs best for deploying in their public outreach. How much more effective is this selected message than the message they would have otherwise used? The answer to this question depends on various parameters whose values remain largely unknown. For example, one such parameter is how much variability there is in message effectiveness across different messages (34). If variability is high, then choosing the top-performing message in a pretest could result in big gains over the business-as-usual message. Whereas, if variability is low, gains may be limited because the “best” messages are only mildly more effective than business-as-usual. Thus, the value of this variability parameter, as well as several others (discussed further below), forms a key assumption governing the potential benefit of survey experiment pretesting.

Furthermore, the above assumptions trade off in ways that are not easily or precisely understood. For example, a campaign may spend 20% of their budget on pretesting, thereby diminishing the number of people it is able to reach with its message. However, if pretesting allows the campaign to identify a sufficiently more effective message, then its diminished reach can be offset by a greater per-person impact among those exposed to it—resulting in more impact overall. On the other hand, if the money spent on pretesting fails to surface a sufficiently more effective message to offset the campaign's diminished reach, then pretesting may harm its impact overall.

In sum, whether or not survey experiment pretesting can increase the impact of public health campaigns in a pandemic depends crucially on assumptions and how they trade off against each other. Yet, we currently lack systematic evaluation of these assumptions and trade-offs, including what existing evidence says (if anything) about the values of key parameters underpinning them.

Therefore, in this paper, we systematically evaluate these assumptions and their trade-offs in a cost-effectiveness analysis of survey experiment pretesting for public health campaigns. Our cost-effectiveness analysis combines (i) a simulation study—in which we systematically vary different assumptions and examine the consequences for campaign impact—with (ii) a review of existing evidence to determine values for the key parameters underpinning those assumptions. Moreover, we anchor our cost-effectiveness analysis to a large published meta-analysis of real public health campaigns that were conducted during the COVID-19 pandemic (35), which examined 376 public health campaigns that ran on Facebook and Instagram between December 2020 and November 2021 and targeted people's attitudes and beliefs about the COVID-19 vaccines. That meta-analysis found that the average campaign spent $105,000, and it estimated a cost of $3.41 per incremental influence on attitudes/beliefs and $5.68 per incremental vaccination for the campaigns.

Anchored to these numbers, the headline results of our cost-effectiveness analysis are as follows.

We estimate that, under reasonable assumptions given the reviewed evidence, survey experiment pretesting is likely cost-effective for public health campaigns with a typical budget of $105,000. Second, and perhaps more importantly, we estimate that pretesting has powerful returns to scale. For well-resourced campaigns (e.g. $210,000+), pretesting is robustly cost-effective on our estimates; on the basis of the above cost per impact numbers, achieving potentially thousands of additional attitudes/beliefs influenced and vaccinations received in expectation. This return-to-scale emphasizes the importance of public health campaigns/organizations sharing resources and findings. Third, our analysis shows how several key parameters powerfully govern whether or not pretesting is cost-effective for campaigns. While evidence to determine the values of these parameters currently exists, it is thin and requires significant improvement. Improving this evidence would provide at least two benefits: (i) determining with greater confidence whether or not (and when) survey experiment pretesting is cost-effective for public health campaigns and (ii) enabling public health campaigns to tailor the size of their survey experiment to maximize its expected benefit. Helpfully, our analysis identifies a specific research design for obtaining evidence on these parameter values—the “parallel megastudy” design—which we describe in detail in the Discussion section.

## Cost-effectiveness analysis

Our cost-effectiveness analysis compares the impact of two hypothetical public health campaigns: one campaign allocates some of its budget for a survey experiment to pretest and choose between different messages for its public outreach (the “experiment campaign”), whereas the other campaign does not (the “no-experiment campaign”). The campaigns are otherwise identical. To be able to make this comparison, our cost-effectiveness analysis consists of three distinct components:

1. Mathematical expressions to calculate the expected impact of each campaign
2. Simulations to estimate the expected improvement in message effectiveness from a survey experiment pretest (informs component #1)
3. A review of existing evidence to estimate key parameter values (informs component #2)

Now, we briefly explain each of these components in turn, using concrete examples. This provides the necessary basis for interpreting the results of our analysis.

### Mathematical expressions to calculate the expected impact of each campaign

To explain this component, let us assume a campaign budget of $105,000, which is the average expenditure of campaigns in the above meta-analysis of real public health campaigns that ran on social media during the COVID-19 pandemic (35). Let us also assume that the cost of producing one message (e.g. a brief video) is $1,000 (for further detail, see the “Description of analysis parameters” section in Materials and methods). The no-experiment campaign produces a message and then spends the rest of their budget disseminating that message. Thus, the expected impact of the no-experiment campaign is:


$$\frac{\mathrm{Budget}-\mathrm{Cost}\;\mathrm{of}\;\mathrm{producing}\;\mathrm{one}\;\mathrm{message}}{\mathrm{Cost}\;\mathrm{per}\;\mathrm{person}\;\mathrm{influenced}}$$

For the denominator, we use the cost of $3.41 per influence on people's attitudes/beliefs, and $5.68 per vaccination, as estimated by the above meta-analysis of public health campaigns (35). Plugging in these numbers implies that the expected impact of the no-experiment campaign is (105,000−1,000)/3.41=30,499 people's attitudes/beliefs influenced and (105,000−1,000)/5.68=18,310 vaccinations received.

What about the expected impact of the experiment campaign? As described above, the expected impact of the experiment campaign depends on the trade-off between three quantities: (i) its budget, (ii) the cost of running the pretest, and (iii) the expected improvement in message effectiveness from running the pretest. Thus, the expected impact of the experiment campaign is given by:


$$\frac{\mathrm{Budget}-\mathrm{Cost}\;\mathrm{of}\;\mathrm{pretest}}{\mathrm{Cost}\;\mathrm{per}\;\mathrm{person}\;\mathrm{influenced}}\;\times \;\mathrm{Expected}\;\mathrm{message}\;\mathrm{improvement}$$

We take the budget and the denominator from above. For the cost of the pretest, let us assume that each survey respondent costs $0.75 and that the experiment campaign conducts a small pretest, producing just two messages and recruiting just 900 respondents (for further detail, see the “Description of analysis parameters” section in Materials and methods). This gives a cost of (1,000 × 2) + (0.75 × 900) = $2,675 for the pretest. Finally, regarding the expected message improvement, for the sake of this example, let us assume the value of this term is 1.10, indicating that the true effect of a message selected via pretesting is 10% more effective in expectation than the average (business-as-usual) message. Plugging in these numbers implies that the expected impact of the experiment campaign is [(105,000−2,675)/3.41]×1.10=33,008 attitudes/beliefs influenced and [(105,000−2,675)/5.68]×1.10=19,816 vaccinations received.

Now, we have calculated the expected impact of both campaigns and can compare them. In this illustrative example, the experiment campaign is expected to influence an extra 33,008 − 30,499 = 2,509 people's attitudes/beliefs, and result in an extra 19,816 − 18,310 = 1,506 vaccinations received, compared with the no-experiment campaign. This equals an 8% increase in expected impact due to the pretesting. Of course, this example is illustrative, and so, we simply picked an arbitrary value for the expected improvement in message effectiveness from running the pretest (i.e. 10%). However, in our analysis, estimating this value is the goal of component #2, described next.

### Simulations to estimate expected improvement in message effectiveness from pretesting

If a campaign tests several different messages and selects the one with largest estimated effect for their public outreach, how much more effective is this selected message than the average (business-as-usual) message in expectation? The answer to this question depends on two sets of parameters.

The first set of parameters concerns the size of the experiment: specifically, how many messages are tested, and with how many survey respondents? The more messages that a campaign produces and tests, the greater chance it can have of testing one that is highly effective. In addition, larger samples of survey respondents will generally result in more precise estimates, meaning that the message with the largest *estimated* effect is more likely to be the message with the *truly* largest effect. If sample size is small, campaigns may often be misled by noise in their message selection.

The second set of parameters concerns the true effects of the messages. There are three parameters to consider. First, since it is easier to detect larger effect sizes than smaller effect sizes, a key parameter in our simulations is the true *average* effect size of messages, which we denote as μ. A second key parameter is the *variability* in message effectiveness across different messages, as discussed earlier. For example, if variability is small, then pretesting can only provide limited gains because the best messages are only mildly more effective than the average message. Following previous work (34), we operationalize variability as the SD in true message effects (τ), normalized by the average message effect (μ); that is, τ/μ. This normalization is necessary because, when we conduct our review of existing studies to inform the values of these parameters (described below), it is common for different studies to use outcome variables with different scales. Thus, the normalization allows us to sensibly aggregate the variability parameter across these different studies. Finally, a third key parameter is the *correlation* between the true message effects in the survey and the true message effects in the “field”—that is, in the actual campaign setting, such as on social media. If this correlation is zero, then the performance of a message in the survey is unrelated to its performance in the field. This would render pretesting useless; the campaign might as well select a message at random from those pretested. We denote this parameter ρ.

In sum, the size of the survey experiment and the true effects of the messages jointly determine the improvement in message effectiveness campaigns can expect from running a pretest. In our analysis, for a given experiment size and configuration of true message effects, we simulate thousands of survey experiment pretests and average across them to obtain the expected true effect of the *selected* message. We then divide this value by the true *average* message effect to calculate the expected improvement over the average message. For example, if the expected true effect of the selected message is 0.11, and the true average message effect is 0.10, then the expected improvement would be 0.11/0.10 = 1.10 = 10% (for further detail, see the “Description of simulations of survey experiment pretests” section in Materials and methods).

Of course, a key uncertainty in our analysis is the values of the parameters just described (μ,τ/μ, and ρ). To inform these values, we thus conducted a review of existing studies, described next.

### Review of existing evidence to inform key parameter values

For brevity, we refer to the “Description of evidence review to inform values of key parameters” section in Materials and methods for full details of our review of existing studies, but briefly summarize below.

To estimate μ, we relied primarily on a 2022 systematic review of survey experiments that tested messages to increase COVID-19 vaccine uptake (36). Estimating τ/μ was more challenging because it required studies that tested more than a handful of different messages—so that we could estimate τ with reasonable precision—as well as access to the raw data so that we could compute the value of τ ourselves (typically studies did not report it). To source studies that met these criteria, we relied on a combination of the aforementioned systematic review (36), snowball sampling, and our knowledge of the literature. Finally, estimating ρ was the most challenging because it required studies that tested the same set of messages in both a survey experiment *and* field experiment—a rare occurrence. We relied on snowball sampling and our knowledge of the literature to source a small handful of studies that offered some evidence as to the potential value of ρ.

Based on our review, we determined values for these parameters that reflect our “best guesses” given the reviewed evidence. However, there is considerable uncertainty in these best-guess values, especially regarding τ/μ and ρ, parameters for which existing evidence is severely limited. Therefore, we also examine a range of other values in our analysis, and we additionally spotlight discrete configurations of parameter values which are more pessimistic and optimistic than the best-guess values. The pessimistic scenario values are smaller than the best-guess values by a factor of two or more, whereas the optimistic values are larger by a factor of 1.5–2. Table 1 displays the parameter values (and their sources) used in our analysis, including those discussed further above. For brevity, for further discussion of the parameter values, we refer to the “Description of analysis parameters” and “Description of evidence review to inform values of key parameters” sections in Materials and methods.

**Table 1.** Parameter values (and their sources) used in our cost-effectiveness analysis.

Parameter	Values	Source
Campaign budget	**Typical: $105k** (Other values: $52.5k, $210k, $420k)	Athey et al. (35)
Cost per message	$1,000	Our review
Cost per survey respondent	$0.75	Our review
Cost per attitude/belief influenced	$3.41	Athey et al. (35)
Cost per vaccination received	$5.68	Athey et al. (35)
Average effect size of messages (μ)	**Best guess: 0.10** Pessimistic: 0.05 Optimistic: 0.20	Our review
Variability in message effects (τ/μ)	**Best guess: 0.40** Pessimistic: 0.20 Optimistic: 0.60 (Other values: from 0.10 to 0.80)	Our review
Survey–field correlation in message effects (ρ)	**Best guess: 0.50** Pessimistic: 0.20 Optimistic: 0.80 (Other values: from 0.10 to 0.90)	Our review

Bolded values are the typical campaign budget and our best guesses about the values of the parameters (based on our evidence review), respectively. We also consider other values in our analysis, as indicated. Average effect size is expressed in standardized units. For further discussion of these parameter values and our evidence review, see the “Description of analysis parameters” and “Description of evidence review to inform values of key parameters” sections in Materials and methods.

## Results

Now, we have explained each component of our cost-effectiveness analysis, we present its results. We begin by presenting results for a typical-sized campaign that has a budget of $105,000.

### Estimated benefit of pretesting for a typical-sized campaign

**Fig. 1.**
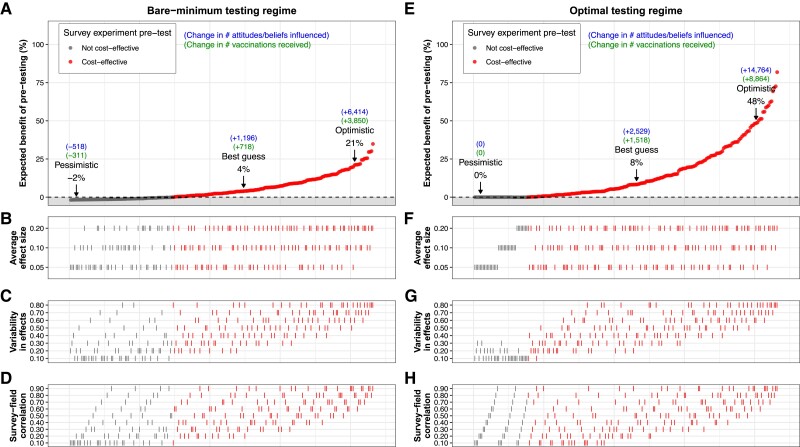
Estimated expected benefit of pretesting for a public health campaign with a budget of $105,000. A–D) The results for the bare-minimum testing regime. E–H) The results for the optimal testing regime. B–D, F–H) The parameter values that correspond to the expected benefit of pretesting in the top panels. There is a clear pattern such that when the variability-in-effects (C, G) and survey–field correlation (D, H) values are larger, pretesting confers a larger expected benefit to the impact of the campaign. Pessimistic, best guess, and optimistic indicate different sets of assumed parameter values (Table 1).

Figure 1 shows the expected benefit of pretesting, estimated by our analysis, across the full range of values we consider for the key parameters above. That is, the average effect size of messages (μ), variability in message effects (τ/μ), and survey–field correlation in message effects (ρ).

There are two sets of results in Fig. 1, representing two different regimes by which the campaign decides on the number of messages and survey respondents to use for its pretest. Under the “bare-minimum” testing regime, the campaign runs a small pretest: producing and testing just two messages with *n* = 900 respondents. In contrast, under the “optimal” testing regime, we assume the campaign has *perfect* knowledge of the true parameter values, and decides on the number of messages and respondents that maximizes the expected benefit of the pretest given these parameter values. For example, if there is large variability in message effectiveness across messages, and the campaign knows this, then it can be worthwhile to produce and test dozens of messages to increase the chance of finding one that is highly effective. The difference in performance between the two testing regimes thus illustrates the benefit of possessing perfect knowledge of the true parameter values (for further details on the optimization procedure, see the “Description of simulations of survey experiment pretests” section in Materials and methods).

Figure 1A–D shows the results under the bare-minimum pretesting regime. Figure 1A shows the estimated expected benefit of pretesting for campaign impact (in %). Scanning across Fig. 1A from left to right, the expected benefit of pretesting ranges from ∼−3% (harm) to as much +35% (large benefit). This range corresponds to different configurations of the parameter values used in our simulations, which are displayed in Fig. 1B–D. There is a clear pattern such that, when the average effect size (Fig. 1B), variability in effects (Fig. 1C) and/or survey–field correlation in effects (Fig. 1D) are larger, pretesting confers a larger expected benefit. This is because the expected improvement in message effectiveness from running a pretest is increasing in these parameter values. For example, when each of these parameters is at the largest value we consider (average effect size = 0.2, variability in effects = 0.8, survey–field correlation in effects = 0.9), the estimated expected benefit of pretesting is at its largest value of +35% (see Fig. 1A–D).

Under our best-guess values for the parameters (Table 1), we estimate that a pretest using the bare-minimum regime increases campaign impact by 4% in expectation. This implies an extra ∼1,200 people's attitudes/beliefs influenced and ∼700 extra people vaccinated due to pretesting (Fig. 1A). However, as described above, there is much uncertainty in this best-guess scenario, because existing evidence for some of the parameter values is limited, and this uncertainty is compounded when considering the parameters together in our analysis. Thus, we also spotlight other configurations of parameter values which are more pessimistic and optimistic than the best-guess values (Table 1). First, consider a more pessimistic set of parameter values. In this case, we estimate that pretesting is *harmful* in expectation, reducing expected impact by −2% (Fig. 1A): the money spent on pretesting fails to surface a sufficiently more effective message to offset the diminished resources for actually disseminating the message, thus harming impact overall. In contrast, under the *optimistic* set of parameter values, we estimate pretesting to be highly beneficial (+21% impact), implying thousands of extra attitudes/beliefs influenced and vaccinations received in expectation (Fig. 1A).

Finally, Fig. 1E–H shows the results of our analysis given an optimal pretesting regime. The pattern of results is qualitatively similar to that of the bare-minimum regime above, except the magnitude of the expected benefit from pretesting is approximately twice as large. This illustrates the advantage of possessing perfect knowledge of the parameter values and tailoring the size of the pretest accordingly. (When the parameter values are sufficiently pessimistic, the optimal decision is to forego pretesting—as a result, the benefit of pretesting is fixed to zero in such cases; see Fig. 1E).

In sum, we estimate that pretesting is likely cost-effective for a typical-sized campaign given our best-guess assumptions about the true values of the parameters and is robustly cost-effective on more optimistic assumptions. Moreover, the estimated benefit is strongly asymmetric across the parameter space: if the true values are on the optimistic side, the benefit could be substantial, whereas if they are on the pessimistic side, the harm appears quite small. Thus, even assuming each of the different parameter scenarios are equally likely, campaigns with a budget of $105,000 would still plausibly benefit in expectation from a pretest. In Supplementary material 1, we show this formally. At the same time, however, it is important to reiterate: if the true parameter values are *in fact* on the pessimistic side, then pretesting (as we conceive of it here) is not cost-effective for a typical-sized campaign. Thus, if public health campaigns are risk-averse—that is, they want to avoid performing worse than if they had foregone any pretesting whatsoever—it is especially important for future research to produce more evidence to determine the true values of the parameters.

### Estimated benefit of pretesting for smaller and larger campaigns

In our analysis thus far, we have focused on a public health campaign with a budget of $105,000, which was the expenditure of a typical campaign in the above meta-analysis of real public health campaigns (35). Now, we consider campaigns with alternative budgets. The SD in campaign budget reported in the meta-analysis was ∼$327,000, implying that some public health campaigns were considerably better funded than others. This raises the important question of how the returns to pretesting scale with the resources available to the campaign.

**Fig. 2.**
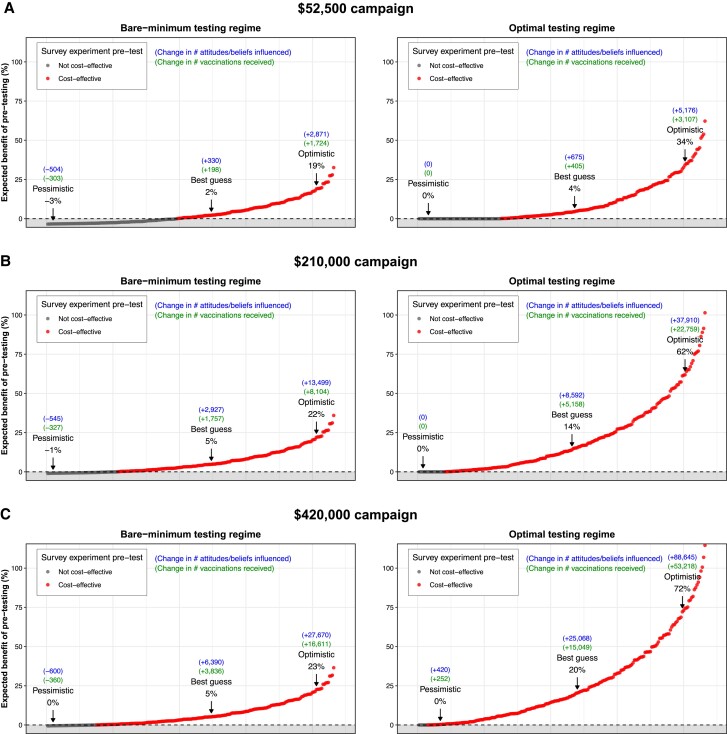
Estimated expected benefit of pretesting for public health campaigns with budgets other than $105,000, using either a bare-minimum or optimal pretesting regime. A) A campaign with budget $52,500; B) a campaign with $210,000; and C) a campaign with $420,000. As with Fig. 1, the value of the estimated expected benefit (*y*-axis) corresponds to different configurations of the key parameter values. However, unlike Fig. 1, to ease interpretation, the lower panels that show the value of those key parameters are omitted in this plot. The full panel plots are reported in Supplementary material 2.

To analyze this question, we repeat our analysis assuming different budgets. Figure 2 shows the estimated expected benefit of pretesting for campaigns with three different budgets: $52,500 (Fig. 2A), $210,000 (Fig. 2B), and $420,000 (Fig. 2C). To ease interpretation, the panels showing the corresponding parameter values are omitted from Fig. 2, but can be viewed in full in Supplementary material 2.

The results shown in Fig. 2 indicate that pretesting has powerful returns to scale. For example, for campaigns with a budget of $210,000 or $420,000, we estimate that pretesting is cost-effective under a wide range of parameter values, increasing campaign impact by 5–20% in expectation under the best-guess parameter values; which in turn implies thousands of extra attitudes/beliefs influenced and vaccinations received (see Fig. 2B and C). The intuition for this return-to-scale result is straightforward: better-resourced campaigns are able to reach more people with their message (by virtue of their extra resources); therefore, uncovering a higher impact message via pretesting acts as a multiplier on this extra reach. An important implication of this return-to-scale result is that, to the extent that public health organizations and agencies can share resources and findings, then the gains to impact from pretesting could potentially be quite large.

### Importance of possessing accurate knowledge of the parameter values

Figures 1 and 2 show that the expected benefit of an optimal pretesting regime, in which campaigns tailor the size of their pretest to the values of the parameters, is considerably larger than that of a bare-minimum pretesting regime (in which the campaign tests just two messages with *n* = 900 respondents only). Therefore, public health campaigns seeking to maximize their impact would presumably want to implement an optimal testing regime for their pretest. However, the performance advantage of the optimal regime depends on perfect knowledge of the true parameter values—knowledge which campaigns does not have in reality. Indeed, in reality, campaigns could optimize their pretesting regime for one set of parameter values when in fact the true values are quite different. In this section, we analyze the consequences of such a mistake.

**Fig. 3.**
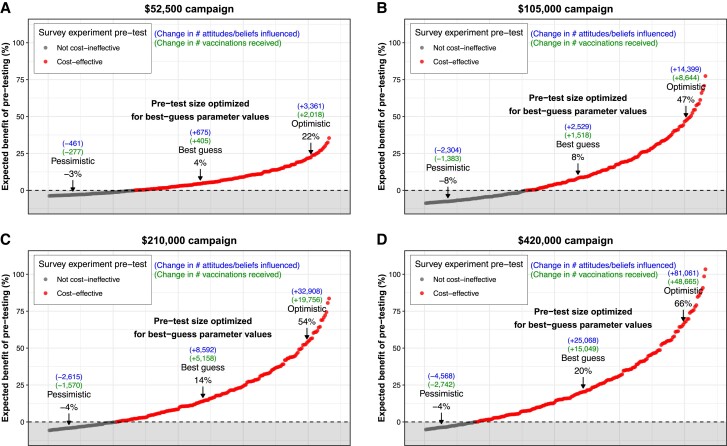
Estimated expected benefit of pretesting when pretest size is optimized for the best-guess set of parameter values. A) The results for a campaign with budget $52,500, B) $105,000, C) $210,000, and D) $420,000. The pattern in each panel shows that when the true parameter values are on the pessimistic side, a campaign that mistakenly optimizes the size of its pretest for the best-guess parameter values ends up harming its impact in expectation. Note that, as with Fig. 2, to ease interpretation, in this plot, we omit the panels that show the value of the key parameters that correspond to the expected benefit. However, these full plots with the lower panels are reported in Supplementary material 3.

Specifically, we estimate the expected benefit of a pretest whose size the campaign optimizes for the best-guess values of the parameters when in reality the true values are different (e.g. more pessimistic or optimistic). For example, for a $105,000 campaign, we determine that the optimal pretest regime under the best-guess parameter values is to produce and test 7 messages with *n* = 4,500 respondents (for further detail on the optimization procedure, see the “Description of simulations of survey experiment pretests” section in Materials and methods). This pretest is much larger than a bare-minimum pretest. If the true parameter values are pessimistic, this could mean that the campaign significantly overspends on its pretest, harming impact. Figure 3 shows the key results of this analysis for campaigns with different budgets.

Figure 3 underscores the importance of possessing accurate knowledge of the parameter values. In particular, while a pretest optimized for the best-guess parameter values increases impact in some areas of the parameter space, in other areas of the parameter space it harms impact. For example, consider a $105,000 campaign: the correctly optimized pretest increases impact by 8% in expectation (Fig. 3B). However, when the pretest is *mistakenly* optimized—for example, if the true parameter values are pessimistic rather than best guess—then the pretest instead harms impact, by −8% (Fig. 3B). Furthermore, this harm is much greater than the harm expected under a bare-minimum pretest (−2%) given the same pessimistic assumptions about the parameter values (see Fig. 1A). This extra harm arises because the mistakenly optimized pretest can be much larger in size (and thus more financially costly) than the bare-minimum pretest. Thus, if the true parameter values are unfavorable for pretesting, most of this money is wasted on pretesting and could instead be better spent on the campaign's public outreach. These results therefore illustrate that, while the gains from optimized-testing can be greater than for a bare-minimum pretest, the losses can also be greater. This underscores the importance of possessing accurate knowledge of the parameter values and for research to produce more evidence to determine their true values.

### Considering the cost of pretest expertise

In a final analysis, we consider the fact that many public health campaigns may not have the expertise and/or infrastructure to conduct survey experiments themselves, and so may need to partner with other actors in order to run a pretest. Such a partnership could impose further financial costs on the campaign, which will reduce any benefit of pretesting. For example, on a consultant model, campaigns could hire an organization with expertise in conducting survey experiments. The cost of this service itself, over and above the cost of the survey respondents (which we already model in our analysis), is difficult to know in general—so we consider a range of additional flat-cost possibilities: $1,000, $3,000, and $10,000. Notably, however, there are alternatives to the consultant model which may be more appealing to public health campaigns.

For instance, the pretest designs considered in our analysis are relatively simple, involving just a handful of different messages and simple random assignment; programming the pretest and analyzing its data are thus relatively straightforward. Indeed, many social and behavioral scientists working in academia regularly perform these tasks as part of their research, and do so with little difficulty. Therefore, an alternative model which is likely more cost-effective for public health campaigns is partnering with academia. Such partnerships could take a variety of forms, and reflect either bespoke one-offs or instead be institutionalized in academic-practitioner networks; an idea that has precedence (37) and was recently recommended by a review of the role of social and behavioral science in the COVID-19 response (38). Such partnerships might involve an agreement whereby, in exchange for running the survey experiments, the academic researchers have the right-to-publish the results in a scientific journal. As well as proving more cost-effective for campaigns, institutionalized partnerships could also act as accelerators for scientific learning: centralizing large amounts of data from experiments testing real public health communication interventions, which researchers could subsequently analyze using meta-analysis. This is a potentially fruitful model for increasing both the impact of public health campaigns, especially in the context of a novel pandemic, as well as scientific understanding, but we leave fleshing it out to future work.

**Fig. 4.**
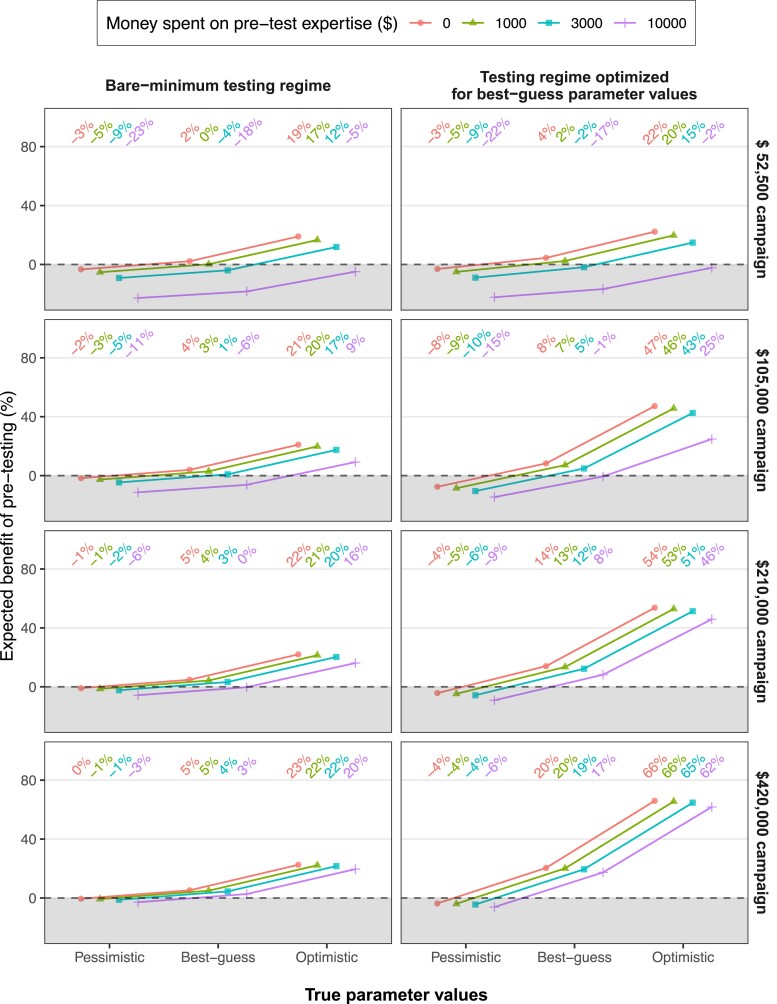
Estimated expected benefit of pretesting given cost of hiring expertise. The plot shows the estimated expected benefit of pretesting as a function of campaign budget (facet rows), pretesting regime (facet columns), true values of the parameters (*x*-axis), and amounts of money spent on hiring pretest expertise (colors). The gray region indicates pretesting is not cost-effective. Unlike Figs. 1–3, this plot only shows the three discrete configurations of the true parameter values (i.e. pessimistic, best guess, and optimistic; see Table 1), rather than the exhaustive combination of all the values we considered in our analysis.

Figure 4 shows the results of our analysis incorporating expertise costs. Specifically, it shows the estimated expected benefit of pretesting for campaigns with different budgets, as a function of their pretesting regime (bare-minimum vs. optimized for best-guess parameter values); the true values of the parameters (pessimistic, best guess, or optimistic); and, crucially, the amount of money spent on hiring expertise to conduct the pretest. The display of Fig. 4 is similar to that of the previous figures, except now there are four curves in each panel—one for each assumed magnitude of expertise cost (i.e. $0, $1,000, $3,000, and $10,000). The results in Fig. 4 show that, for a typical-sized campaign (budget $105,000), pretesting plausibly remains cost-effective under small ($1,000) and moderate ($3,000) expertise costs. However, under large expertise costs ($10,000), it does not. In contrast, for campaigns with larger budgets, pretesting likely remains cost-effective even under large expertise costs. Finally, campaigns with smaller than average budgets ($52,500) can only tolerate small expertise costs before pretesting is no longer cost-effective on our analysis.

## Discussion

Following the COVID-19 pandemic, the World Health Organization identified public engagement and communication one of the several areas requiring further research and development to improve society's response to future pandemics (5) (see also Ref. (15)). In this paper, we aimed to respond to this call by conducting a cost-effectiveness analysis of survey experiment pretesting as a tool that public health campaigns could use to potentially increase their impact during a pandemic. Given the reviewed evidence, our analysis suggests that pretesting could likely increase the impact of public health campaigns in a pandemic context, particularly if campaigns are well-resourced and/or can draw upon resources and findings shared across public health organizations.

A core contribution of our analysis is to quantify the key parameters that govern the benefit of pretesting for public health campaigns; namely, the variability in message effects, and the correlation between survey effects and those effects in the field (i.e. in an actual campaign setting). The values of these parameters powerfully determine the increase in impact that is possible from survey experiment pretesting. Moreover, we show that possessing good information about the values of these parameters allows the size of the pretest to be optimized, potentially enabling further increases in impact. While there is some existing evidence that can speak to the true values of these parameters, it remains limited (see the “Description of evidence review to inform values of key parameters” section in Materials and methods). To improve this evidence, future research in public health communication can use a new study design: the parallel megastudy design. This design combines two existing designs, the in-survey megastudy (39, 40) and the in-field megastudy (41, 42) both of which involve testing many different interventions simultaneously. In the parallel megastudy design, an in-survey and in-field megastudy are conducted *in parallel* using the same set of messages. As a result, the parallel megastudy design allows researchers to estimate both the variability in message effects and the correlation between survey and field effects. The parallel megastudy design can thus rapidly advance scientific understanding of the potential benefit of survey experiment pretesting for public health campaigns.

Our results are anchored to the details of hundreds of public health campaigns that ran on Facebook and Instagram aiming to encourage vaccination during the COVID-19 pandemic (35), as well as to an evidence review that drew primarily on studies of messages encouraging COVID-19 vaccination. Therefore, our results would likely generalize most readily to similar contexts; that is, vaccination campaigns conducted on social media during a public health emergency. Our results of course also highlight the possibility that campaigns whose goal is to encourage vaccination for nonpandemic diseases (e.g. flu, Human Papillomavirus) or other health behaviors (e.g. contraceptive use, clinic visits) could similarly increase their impact via pretesting. However, it is important to recognize the significant challenges confronting such generalization, even to contexts that are most similar to the one we study here. To take one salient example, public health emergencies of the future are likely to be different from the COVID-19 pandemic in myriad ways—not only regarding the features of the disease itself (e.g. severity, transmissibility), but also in background political conditions, trust (or distrust) toward health institutions, the types of social media with which people engage, and so on. These contextual/setting-based factors are a key dimension that can limit generalization, in addition to various other factors like the population and outcome under study (28, 43, 44). Thus, our results should be generalized with caution, especially when the target context is expected to differ considerably from that which we study here, and our analysis is certainly not the final word on the potential efficacy of survey experiment pretesting for public health campaigns.

Now, we highlight some additional limitations of our analysis and results.

First, readers should not mistake potentially large *relative* increases in campaign impact from pretesting (e.g. 20%) for large *absolute* increases in campaign impact. On the contrary, our analysis shows that, even for the best-resourced campaigns operating under optimistic assumptions, the expected increase in impact from pretesting is limited to tens of thousands of additional attitudes/beliefs influenced and vaccinations received. In other words, even in the best case, it is clear that survey experiment pretesting could only form a small part of a successful pandemic response. Indeed, more broadly, we concur with other scholars that the potential impact of interventions encouraging individual-level behavior change—such as public health campaigns—typically comes a distant second to the impact of policy- and other system-level interventions (45). While interventions encouraging individual-level behavior change can cost-effectively contribute to a successful outcome, this asymmetry in impact should be kept in mind (46).

Second, our analysis does not detract from the importance of conducting field experiments for evaluating the impact of public health campaigns. While our results suggest that survey experiment pretesting can increase the impact of such campaigns, conducting field experiments is necessary to understand the magnitude of the campaigns’ impact in the real world, and whether it is worth the cost of running such campaigns at all. If sufficiently few people are willing or able to watch a public health campaign's video on social media, for example, then money may be better spent elsewhere.

Third, to compute the additional numbers of vaccinations expected due to pretesting, our analysis relied on the cost per incremental vaccination estimated by Athey et al. (35) in their meta-analysis of COVID-19 social media campaigns. Their estimate assumes that changing people's self-reported attitudes, beliefs and/or intentions to get vaccinated converts to actual vaccinations at a rate of 0.6. In other words, if 10 people were to report being in favor of the COVID-19 vaccinations when previously they were opposed, we should expect six of them to actually get vaccinated. This discount rate represents the well-known “intention-behavior gap” (47, 48). If the discount rate is <0.6, then our estimates of the number of additional vaccinations should also be correspondingly shrunk. Notably, estimates of the discount rate reported in other research studies (conducted in various different contexts) are between 0.33 and 0.55 (47–50).

Fourth, another assumption in our analysis for which there is currently limited evidence is that the variability in message effects in the field (campaign setting) is *proportional* to the variability in effects in the survey (despite the absolute effect sizes are much smaller in the field, as expected). If the variability in effects in the field is proportionally larger than in the survey, our analysis will underestimate the benefit of pretesting; if the reverse is true, our analysis will overestimate its benefit. Notably, the parallel megastudy design we describe above can also bring evidence to bear on this question, further highlighting the value of this design for advancing scientific understanding of the returns to survey experiment pretesting for public health campaigns.

To conclude, we reiterate that various sources suggest the next pandemic is a question of “when” not “if” (2–6). Understanding how the social and behavioral sciences can contribute to a successful pandemic response is a worthwhile goal—one that we aimed to advance here.

## Materials and methods

### Description of analysis parameters

#### Campaign budget

As described in the main text, in order to choose campaign budgets for our analysis, we referred to a large meta-analysis of 376 real public health campaigns that ran on social media through 2021 that targeted people's beliefs/attitudes about the COVID-19 vaccines (35). The average campaign spend was ∼$105,000, with a SD of ∼$327,000, implying that some campaigns had substantially larger budgets than average. Thus, we consider a range of budgets.

#### Cost per message

There are various formats that public health campaigns could use to disseminate their message, with plausibly different production costs for the messages. In line with our focus on the aforementioned meta-analysis of COVID-19 social media campaigns, we assume the messages are brief social-media-style videos. The cost of producing one such video is likely to depend upon where it is commissioned. According to the popular marketplace Upwork (https://www.upwork.com/), many “social media videographers” charge between $50 and $100 per hour. Therefore, we assume a per-video cost of $1,000, which translates to 10–20 h of work by a social media videographer.

#### Cost per survey respondent

For the cost per survey respondent, we refer to popular survey providers used by behavioral scientists, such as Prolific (https://www.prolific.co/) and Cloud Research (https://www.cloudresearch.com/). For a 3-min survey that pays $11 per hour, the cost per survey respondent on prolific is ∼$0.75, inclusive of their service fee (on Cloud Research, the figure is similar). We thus assume a cost of $0.75 per respondent. Notably, this cost is for a convenience sample not a national probability sample.

#### Cost per attitude/belief influenced and vaccination received

These parameters are explained in detail in the main text; thus, we refer readers there. Their values are taken from a meta-analysis of real public health campaigns from the COVID-19 pandemic (35).

#### Cost of pretest expertise

This parameter is explained in detail in the main text; thus, we refer readers there. Note that, for the results presented in Figs. 1–3, the expertise cost is set as $0.

#### Average effect size of messages

This parameter refers to the true (i.e. latent) mean effect size in the survey environment, not the estimated mean effect size in a particular survey experiment. This distinction is important insofar as the estimated mean effect size in a particular survey experiment need not equal the true mean effect size, due to sampling variability. We consider values of 0.05, 0.1, and 0.2 standard units, which are all considered small or negligible effect sizes by conventional academic standards (51). In the “Description of evidence review to inform values of key parameters” section in Materials and methods, we describe the evidence that informs these values.

#### Variability in message effects

This parameter refers to the true variability in effect sizes across messages—indicated by the SD (τ)—normalized by the effect size of the average message (***µ***). This normalization follows the approach taken by previous work (34) and is convenient in our case because it allows us to compare and aggregate the variation in effect sizes estimated in different studies (described later in the “Description of evidence review to inform values of key parameters” section in Materials and methods). We consider values that range from 0.1 to 0.8 in increments of 0.1. A value of 0.1 implies that one SD in message effects is equal to one-tenth of the average message effect; that is, it implies the messages barely vary from the average effect. In contrast, a value of 0.8 implies that one SD in message effects is equal to four-fifths of the average message effect; and, thus, that we should expect reasonably large variability in effectiveness across different messages. In the “Description of evidence review to inform values of key parameters” section in Materials and methods, we describe evidence that informs the value of this parameter.

#### Survey–field correlation in message effects

This parameter refers to the correlation between the true effects of messages in the survey environment and the true effects of those messages in the field (i.e. an actual campaign setting).

There are several reasons why this correlation may be <1. To pick just one example, there may be between-person heterogeneity in the effects of different messages. For instance, among highly educated respondents, message *X* is more effective than message *Y*, whereas among less educated respondents the reverse is true. If the survey sample of respondents does not represent the target population for the campaign (e.g. highly educated respondents are overrepresented in the survey), then the most effective message in the survey may not be the most effective message in the field, thereby diminishing the survey–field correlation in message effects. Notably, evidence suggests that between-person heterogeneity in message effects tends to be small (52, 53), though even small differences could still have important implications for campaign impact (54).

We consider a range of values for the survey–field correlation: from 0.1 to 0.9, in increments of 0.1. In the “Description of evidence review to inform values of key parameters” section in Materials and methods, we describe evidence that informs the value of this parameter.

### Description of simulations of survey experiment pretests

For each unique set of parameter values, we simulate 3,000 survey experiment pretests. As described in the main text, the purpose of these simulations is to estimate the expected improvement in message effectiveness from running the pretest and selecting the message with the largest estimated effect. The procedure for each of the simulated survey experiments is as follows.

First, we draw *X* messages from a bivariate Gaussian distribution, where *X* is an integer between 2 and 20. The two dimensions of the bivariate distribution correspond to the true message effects in-survey vs. the true effects of the messages in the field. The distribution has a mean, equal to the average effect size of messages, and a covariance matrix that captures (i) the variability in the message effects and (ii) the survey–field correlation between the effects.

We then randomly assign each of *Y* simulated respondents to one of the messages, where *Y* is an integer between 500 and 20,000. We also assign some of the simulated respondents to a control group, to account for the fact that it is likely campaigns would include a control group (that receives no message) to check that their messages have an effect in the intended direction. Specifically, in expectation each message group is assigned *Y*/(*M* + 1) respondents, where *M* is the number of messages in the simulation. After respondents have been assigned, we add noise to the true effect of each message to simulate sampling variability. The noise assumes that 10% of the variance in the outcome can be explained by adjusting for pretreatment covariates (e.g. age, gender, etc.).

In the final step, we identify the message with the largest estimated effect in the survey. Then, we assume that the message that gets selected on the basis of the survey pretest exerts an effect in the actual campaign that is equal to its true in-field effect size. This follows previous work (34).

After 3,000 simulated pretests, we take the mean of the 3,000 selected messages’ true in-field effects, and divide this number by the average effect size of messages. This ratio therefore tells us the expected improvement in message effectiveness from performing the pretest. For example, if the ratio is 1.1, this tells us that the pretest procedure identifies messages that are 10% more effective than the average, business-as-usual message in expectation.

For the bare-minimum testing regime, the number of respondents and messages tested is always the same (*n* messages = 2 and *n* respondents = 900). Whereas, for the optimal testing regime, for each unique combination of parameter values we perform a grid search over the joint distribution of *n* messages (2–20) and *n* respondents (500–20,000) to find the combination of messages and respondents that maximizes the expected benefit of pretesting. After finding the optimal combination, we re-estimate the expected benefit using this combination in order to avoid the winner's curse inflating its benefit. If the expected benefit is negative, we assume the campaign (correctly) decides not to run a pretest; in such cases, the expected benefit is simply fixed to zero.

### Description of evidence review to inform values of key parameters

In this section, we describe our review of existing evidence to inform the best guess, pessimistic, and optimistic configurations of the key parameter values μ, τ/μ, and ρ.

#### Average effect size of messages (μ)

To inform plausible values for this parameter, we relied primarily on a 2022 systematic review of randomized controlled trials (RCTs) that evaluated interventions to increase COVID-19 vaccine uptake (36). We examined all of the studies in that review that used survey experiment to evaluate one or more messages on people's self-reported attitudes, beliefs, and/or behavioral intentions related to the COVID-19 vaccines. This amounted to 25 studies. To these 25 studies, we added a further 11 studies, all of which focused on COVID-19 outcomes, as well as 1 meta-analysis of public health communication on various non-COVID outcomes. These extra studies were identified using snowball sampling and our knowledge of the literature. We therefore examined 37 effect sizes in total.

For each study, we extracted the average effect size across the messages and, where necessary, standardized the effect size by dividing by the SD of the outcome variable. In many cases, this required a back-of-the-envelope calculation. For example, some studies reported their effect sizes in percentage points, but did not report the SD of the outcome variable, meaning we could not calculate the standardized effect size directly. In cases like this, we took a maximally conservative approach and used the SD of a uniform distribution over a 0–1 binary scale (equal to 0.5), ensuring that we would err on the side of underestimating the standardized effect size (Table S1 provides further detail about the studies and our calculations). In addition, some of the studies lacked a “pure” control group that received no relevant information; instead, people in the control group received baseline relevant information. This also renders our standardized effect size estimate conservative. Some studies did not report the actual point estimates of the message effects—opting to display them in figures only—so we approximated the estimates based on the figures. Finally, where studies included multiple relevant outcome variables, we took the mean across the estimated message effects for each outcome.

Across the 37 extracted effect sizes, the mean standardized effect size is 0.12 and the median is 0.08. Notably, we do not compute a precision-weighted average because one of the studies 49 has a sample size (∼484,000) that is several orders of magnitude larger than any of the others. It is desirable to avoid letting that study dominate the average effect size here because that would unduly privilege the very specific outcome variable and intervention type used in that study over the many possible outcome variables and intervention types. In sum, therefore, on the basis of this evidence, we assume a best-guess mean effect size of 0.1 standard units. For the pessimistic and optimistic values, we halve and double the best-guess value, respectively, giving values of 0.05 (pessimistic) and 0.20 (optimistic). These effect sizes are typically considered small or even tiny (51).

#### Variability in message effects (τ/μ)

Evidence for this parameter must meet demanding criteria. Specifically, to be informative for the context in which campaigns would actually perform a pretest, the message effects must be estimated on (i) the same outcome variable, (ii) the same sample of people, and (iii) under broadly similar background conditions. This rules out meta-analyses of public health communication (and meta-analyses of other types of communication), since meta-analyses nearly always combine studies which differ from one another along one of the aforementioned dimensions. Generally, these differences will inflate the estimated variability in message effects because some outcome variables or types of people are more receptive to interventions than are others. Thus, excluding meta-analyses renders our estimate of the variability in message effects conservative. In short, to obtain estimates of the variability that are relevant for the context in which campaigns would perform a pretest, the effects of multiple different messages must be estimated within the same study.

In addition, because our quantity of interest is the variability across message effects, studies that used just a small sample of messages can only offer highly uncertain estimates of this variability (an analogy is estimating the SD of a variable for which there are only several data points, each of which is itself measured with uncertainty). For this reason, studies should estimate the effects of more than a handful of different messages, ideally many more. Such studies will typically demand large sample sizes of survey respondents. Lastly, even when such studies have been conducted, they must have actually reported an estimate of the variability in message effects.

We could not locate any studies of public health communication that met all these criteria. Thus, for the current paper, we sought to identify relevant studies and re-analyze their data ourselves in order to estimate the variability in message effects. To that end, we drew on the above systematic review of COVID-19 interventions (36), as well as snowball sampling and our knowledge of the literature, to identify 14 studies of public health communication, each of which investigated at least 5 different messages. Of these studies, one did not contain a control group and so could not be included in our re-analysis (a control group is necessary to estimate each of the message effects). Another study included some messages that tried to *dis*courage vaccination; this study was also excluded from our re-analysis as it had fewer than five messages aimed at encouraging vaccination. A further two studies we identified used highly overlapping data, so we only included the data from one study in our re-analysis. Finally, while some of the studies had publicly available data, many did not, and we could not access the data of one study despite contacting the listed corresponding author. This left 10 studies whose data we re-analyzed (detailed in Table S2).

We used the following strategy to estimate the variability in message effects for each study. First, we computed the average treatment effect of each message relative to the study's control group. We then conducted a random-effects meta-analysis (55) of the message effects for a given study, which provided an estimate of: (i) the SD in message effects (τ), taking into account the error with which each message effect is estimated, as well as (ii) the mean message effect (μ). Finally, for each study, we normalized τ by dividing it by μ, (i.e. τ/μ), thereby allowing us to aggregate the estimated variability across studies. Without this normalization, the τ estimates are not comparable across studies because different studies use different outcome variables, and some outcomes could have larger estimated τ simply because of their scale. If a study included multiple outcomes, we conducted the above procedure for each outcome and then computed the mean τ/μ across outcomes. Further details about these analyses are reported in Table S2.

Across the 10 re-analyzed studies, the mean τ/μ is 0.59 and the median is 0.27. That is, across these studies, one SD in message effects is estimated to be equal to between one-quarter and three-fifths of the size of the average message effect. However, as expected, there is substantial heterogeneity in τ/μ across studies (see Table S2). This is likely due in part to the small numbers of messages in each study; the median number of messages investigated in the studies was just seven. Thus, the estimates of τ/μ are likely to be highly heterogeneous between studies due to sampling variability, and, as a result, the average τ/μ across studies is correspondingly uncertain. In addition to this, most of the studies examined messages that were simply static lines of text. However, in many public health communication contexts, such as the COVID-19 campaigns that ran on social media (35), the messages are likely to be professionally produced videos. This is relevant because the true variability in message effects may be larger when the messages in question differ not only in their text content but also in their video and audio content.

To account for the small samples of messages and lack of video/audio content in each study, we supplemented our re-analysis with three additional studies from the domain of political communication, sourced via our knowledge of the literature (34, 53, 56). The details of these studies are also reported in Table S2. Importantly, these additional studies had unusually large samples of messages—the median number of messages investigated was 59—and the messages in each study were all short videos with audio content. Furthermore, each of these studies already reported an estimate of τ and μ, obviating the need for us to re-analyze their data. The mean value of τ/μ across these three studies is 1.23 and the median is 0.95. Thus, while these studies should receive less weight than the already-analyzed studies given that their focus is political communication rather than public health communication, their estimates suggest that variability in message effects may indeed be larger when messages differ in video/audio content.

Taking this evidence together with our estimate of the average variability in message effects across the studies of public health communication re-analyzed earlier (i.e. mean τ/μ=0.59, median τ/μ=0.27), we settle on a best-guess value of 0.4 for the variability parameter overall. A value of 0.4 implies that the true SD in message effects is equal to two-fifths of the effect size of the average message. For example, if the effect size of the average message (μ) is 0.1 standard units, a variation parameter of 0.4 implies that τ = 0.4 × 0.1 = 0.04. This in turn implies that most message effects will fall between 0.06 and 0.14 (i.e. 0.1 ± 0.04); that is, most messages will have true effects that are small by conventional academic standards and that are not too dissimilar to the average message effect. For the pessimistic and optimistic values of this parameter, we halve and 1.5× the best-guess value, respectively, giving values of 0.2 (pessimistic) and 0.6 (optimistic).

#### Survey–field correlation in message effects (ρ)

Evidence for this parameter is the most demanding of all the parameters we consider in our analysis. To estimate the correlation between the effects of messages in-survey and those same messages in the field, relevant studies are those that have conducted a survey and field experiment in parallel using the same set of messages—and ideally using a large sample of messages to minimize sampling variability. Because field experiments that study a large number of messages are resource-intensive, the pool of potentially relevant studies is small at the outset. Moreover, prominent field experiments that included a large number of public health messages (27, 57, 58) typically did not conduct a parallel survey experiment and, as a result, cannot estimate the survey–field correlation.

**Fig. 5.**
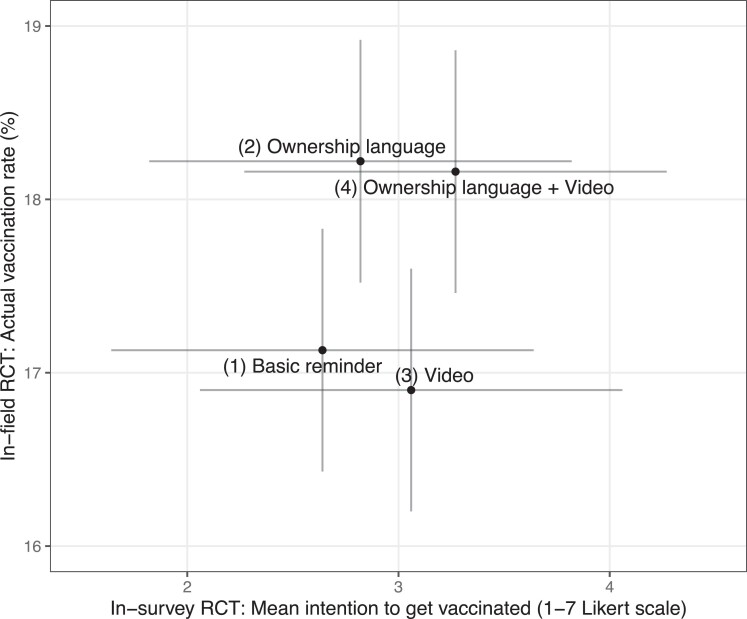
Message effects from survey experiment and field experiment reported by Dai et al. (33). Note that the displayed CIs are approximate (see in text).

We located just one study of public health communication that conducted a field and survey experiment in parallel, using a set of four messages (33). In that study, respondents in the field experiment were randomized to receive via cell phone text one of the four different treatment messages encouraging them to schedule an appointment for their COVID-19 vaccination. The messages consisted of either (#1) a basic reminder, (#2) a reminder that used “ownership” language, (#3) a basic reminder with video content, or (#4) the ownership reminder with the video content. The *y*-axis of Fig. 5 shows the estimated vaccination rate in each of these message groups from the field experiment (copied from Fig. 2b in Ref (33)); the *x*-axis shows people's self-reported vaccination intentions in each of the message groups from the corresponding survey experiment in which vaccination intentions were measured (from Fig. 5b in the supplement of Ref. (33)). We approximate CIs from visual inspection of the SEs because we could not locate the SEs/CIs on the means reported in numeric form.

Figure 5 could be interpreted as providing evidence against a positive correlation between survey and field message effects because, in the survey RCT, the messages with the video content performed better on average, whereas those with the ownership language did not. In contrast, in the field RCT, the reverse pattern was observed. Nevertheless, closer inspection of the results suggests this interpretation is not quite right. In particular, in the survey RCT the top-performing message on respondents’ vaccination intentions was message #4. In the field RCT, this message was also a top performer on increasing vaccination rates; its point estimate was close to that of the “winning” message (#2). The implication is that, had a public health campaign selected the top-performing message from the survey RCT (#4) for running in their outreach to increase vaccination uptake, it would have been a reasonably good choice. In contrast, had they tried to infer a general principle of message development—such as “video content performs best”—they would have been misled. But, recall that the goal of in-survey RCT pretesting (as operationalized in the current article) is not to infer such principles; rather, it is to select the top-performing individual message from among several different messages tested. Added to this interpretational ambiguity, furthermore, is the fact that the evidence in Fig. 5 for the value of the survey–field correlation is extremely limited by the small set of only four messages—all of whose effects are estimated with relatively large amounts of noise. As a result, we are cautious to conclude much from this evidence about the likely value of the survey–field correlation parameter.

In an effort to gather more evidence, we looked to larger studies conducted outside the domain of public health communication (25, 59, 60). These studies point toward a moderate positive survey–field correlation. We briefly describe them below.

Hainmueller et al. (59) studied data from Switzerland in which some municipalities used referendums to vote on the naturalization applications of immigrants. In the referendums, voters received a leaflet with a short description of the applicant, including information about their attributes, such as age, education, and so on, and then cast a secret ballot to accept or reject individual applicants. Voters decided over thousands of immigrants with varying characteristics, allowing identification of how much each particular attribute affected the probability of being accepted by voters in a real-world setting. Ten years later, the authors conducted survey experiments in which survey respondents completed a hypothetical referendum task, choosing whether to accept or reject hypothetical immigrant profiles based on similar attributes as in the real referendums. The authors then used estimates of attribute importance from their survey data to generate predicted probabilities of acceptance for each of the immigrant applications from the real referendums. These survey-based predictions were correlated at 0.5 (on average) with the probabilities of acceptance for each application generated by the model that was fitted to the actual referendum data. In other words, the survey-based estimates were correlated with the field-based estimates at an average of 0.5, even despite a 10-year gap between the two sets of estimates.

Another piece of evidence comes from Coppock and Green (60), who examined paired survey and field effect sizes from 12 different studies of political behavior phenomena. They estimated an overall rank-order correlation of 0.73 between the pairs of estimates.

A final piece of evidence comes from O’Keefe (25). One mechanism through which in-survey message effects may be poorly correlated with field effects is that the outcome variable in a survey is typically self-reported (e.g. behavioral intentions) whereas the outcome in the field is the actual behavior. O’Keefe conducted a meta-analysis of 317 studies in which 2 messages were compared (e.g. a loss-framed message vs. a gain-framed message) on different outcome variables: self-reported outcomes (intentions, attitudes) and behavioral outcomes. He examined how often the direction of the difference between messages was the same on the different types of outcomes, such as the loss-framed message having a larger effect than the gain-framed message on behavioral intentions as well as on behavior. He found that in 82% of possible comparisons (49/60), the direction of the difference between messages was the same on the attitude outcome as it was on the behavioral outcome; while, in 94% of possible comparisons (102/109), the direction was the same on the behavioral intention outcome as it was on the behavioral outcome.

These percentages (82%, 94%) imply correlations of at least 0.85 between the message effects estimated on the self-reported outcomes and those estimated on the behavioral outcomes. To see this, we conducted a simple simulation in which we sampled two “messages” from a bivariate normal distribution, and selected the message with the highest value on the first dimension. We then recorded whether this was also the message with the highest value on the second dimension; that is, whether the rank order of the message values was the same on both dimensions. When the true correlation between messages is set to 0.85, the sampled messages have the same rank order ∼82% of the time. Thus, the O’Keefe study suggests that the survey–field correlation is not dramatically attenuated by the fact that survey experiments rely on self-reported outcomes.

In sum, there is very limited evidence regarding the value of the survey–field correlation in message effects. However, the little evidence that does exist is either inconclusive or points toward at least a moderate or even strong correlation. Thus, considering the evidence together, we settle on a best-guess correlation of 0.5 between in-survey and in-field message effects for our context. To give an intuitive sense of what this means, a correlation of 0.5 implies that, if a campaign were to correctly identify the best of 2 different messages in a survey experiment, that message would also be the best message in the field ∼66% of the time (this percentage is determined using the same simulation approach as described in the previous paragraph). Reflecting the limited evidence, we use wide pessimistic and optimistic values for the correlation: 0.2 and 0.8, respectively. Finally, we note that references [61–68] are referred to from the Supplementary material.

## Supplementary Material

pgae469_Supplementary_Data

## Data Availability

The code to reproduce the results reported in this paper can be downloaded from https://osf.io/k597m/. The remaining data needed to evaluate the conclusions in the paper are present in the paper and the Supplementary material.

## Preprint

This manuscript was posted on a preprint server: https://osf.io/9nyze/.

## References

[1] Our World in Data. Estimated cumulative excess deaths during COVID, *Our World in Data*. [accessed 2024 Mar 28]. https://ourworldindata.org/grapher/excess-deaths-cumulativeeconomist-single-entity.

[2] Juan C . 2023. What Comes After COVID—Asterisk. [accessed 2024 Mar 28]. https://asteriskmag.com/issues/02/what-comes-after-covid.

[3] Marani M, Katul GG, Pan WK, Parolari AJ. 2021. Intensity and frequency of extreme novel epidemics. Proc Natl Acad Sci U S A. 118:e2105482118. 34426498 10.1073/pnas.2105482118 PMC8536331

[4] Inglesby T . 2023. Opinion | How to Prepare for the Next Pandemic, *The New York Times*. [accessed 2024 Mar 28]. https://www.nytimes.com/2023/03/12/opinion/pandemic-health-prepare.html.

[5] World Health Organization . 2022. How global research can end this pandemic and tackle future ones. [accessed 2024 Mar 28]. https://www.who.int/publications/m/item/how-global-research-can-end-this-pandemic-and-tackle-future-ones.

[6] UK Government . 2023. National Risk Register 2023”. [accessed 2024 Mar 28]. https://assets.publishing.service.gov.uk/government/uploads/system/uploads/attachment_data/file/1175834/2023_NATIONAL_RISK_REGISTER_NRR.pdf.

[7] No government can address the threat of pandemics alone—we must come together, *GOV.UK*. [accessed 2024 Mar 28]. https://www.gov.uk/government/speeches/no-government-can-address-the-threat-of-pandemics-alone-we-must-come-together.

[8] Vora NM, et al 2022. Want to prevent pandemics? Stop spillovers. Nature. 605:419–422. 35551284 10.1038/d41586-022-01312-y

[9] Dzau V, Yadav P. 2023. The influenza imperative: we must prepare now for seasonal and pandemic influenza. Lancet Microbe. 4:e203–e205. 36738755 10.1016/S2666-5247(23)00013-7 PMC9891732

[10] James E . 2021. R-I. Sen. Risch, S.2297–117^th^ Congress (2021–2022): International Pandemic Preparedness and COVID-19 Response Act of 2021. [accessed 2024 Mar 28]. http://www.congress.gov/.

[11] The 80,000 Hours Team. Preventing catastrophic pandemics, *80,000 Hours*. [accessed 2024 Mar 28]. https://80000hours.org/problem-profiles/preventing-catastrophic-pandemics/.

[12] Betsch C, et al 2022. A call for immediate action to increase COVID-19 vaccination uptake to prepare for the third pandemic winter. Nat Commun. 13:7511. 36473855 10.1038/s41467-022-34995-y PMC9726862

[13] Nature Outook. Pandemic preparedness, *Nature*. 2022. [accessed 2024 Mar 28]. https://www.nature.com/collections/jaacfgeief.

[14] Coalition for Epidemic Preparedness Innovations . New vaccines for a safer world. [accessed 2024 Mar 28]. https://cepi.net/.

[15] Nuzzo JB, Ledesma JR. 2023. Why did the best prepared country in the world fare so poorly during COVID? J Econ Perspect. 37:3–22.

[16] Chandler J, Shapiro D. 2016. Conducting clinical research using crowdsourced convenience samples. Annu Rev Clin Psychol. 12:53–81. 26772208 10.1146/annurev-clinpsy-021815-093623

[17] Peyton K, Huber GA, Coppock A. 2022. The generalizability of online experiments conducted during the COVID-19 pandemic. J Exp Polit Sci. 9:379–394.

[18] Chandler J, Rosenzweig C, Moss AJ, Robinson J, Litman L. 2019. Online panels in social science research: expanding sampling methods beyond Mechanical Turk. Behav Res Methods. 51:2022–2038. 31512174 10.3758/s13428-019-01273-7 PMC6797699

[19] Fowler C, Jiao J, Pitts M. 2022. Frustration and ennui among Amazon MTurk workers. Behav Res Methods. 55:3009–3025. 36018485 10.3758/s13428-022-01955-9 PMC9415248

[20] Robinson J, Rosenzweig C, Moss AJ, Litman L. 2019. Tapped out or barely tapped? Recommendations for how to harness the vast and largely unused potential of the Mechanical Turk participant pool. PLoS One. 14:e0226394. 31841534 10.1371/journal.pone.0226394 PMC6913990

[21] Coppock A, McClellan O. 2019. Validating the demographic, political, psychological, and experimental results obtained from a new source of online survey respondents. Res Polit. 6:2053168018822174.

[22] Shen F, Sheer VC, Li R. 2015. Impact of narratives on persuasion in health communication: a meta-analysis. J Advert. 44:105–113.

[23] O’Keefe DJ, Hoeken H. 2021. Message design choices don’t make much difference to persuasiveness and can’t be counted on—not even when moderating conditions are specified. Front Psychol. 12:664160. 34267703 10.3389/fpsyg.2021.664160 PMC8275937

[24] Bavel JJV, et al 2020. Using social and behavioural science to support COVID-19 pandemic response. Nat Hum Behav. 4:460–471. 32355299 10.1038/s41562-020-0884-z

[25] O’Keefe DJ . 2021. Persuasive message pretesting using non-behavioral outcomes: differences in attitudinal and intention effects as diagnostic of differences in behavioral effects. J Commun. 71:623–645.

[26] Dimant E, Clemente EG, Pieper D, Dreber A, Gelfand M. 2022. Politicizing mask-wearing: predicting the success of behavioral interventions among republicans and democrats in the U.S. Sci Rep. 12:7575. 35534489 10.1038/s41598-022-10524-1 PMC9082983

[27] Milkman KL, et al 2022. A 680,000-person megastudy of nudges to encourage vaccination in pharmacies. Proc Natl Acad Sci U S A. 119:e2115126119. 35105809 10.1073/pnas.2115126119 PMC8833156

[28] Druckman JN . 2021. A framework for the study of persuasion. Annu Rev Polit Sci. 25:2.1–2.24.

[29] Blumenau J, Lauderdale BE. 2021. The variable persuasiveness of political rhetoric. Am J Polit Sci. 68:255–270.

[30] Rode JB, et al 2021. Influencing climate change attitudes in the United States: a systematic review and meta-analysis. J Environ Psychol. 76:101623.

[31] Bowen D . 2022. Simple models predict behavior at least as well as behavioral scientists. arXiv, arXiv:2208.01167. preprint: not peer reviewed.

[32] Broockman D, Kalla J, Caballero C, Easton M. 30 October 2023. Political practitioners poorly predict which messages persuade the public. OSF 8un6a. 10.31219/osf.io/8un6a, preprint: not peer reviewed. PMC11551421 39467135

[33] Dai H, et al 2021. Behavioural nudges increase COVID-19 vaccinations. Nature. 597:404–409. 34340242 10.1038/s41586-021-03843-2 PMC8443442

[34] Hewitt L, et al 2024. How experiments help campaigns persuade voters: evidence from a large archive of campaigns’ own experiments. Am Polit Sci Rev. 1:1–19.

[35] Athey S, Grabarz K, Luca M, Wernerfelt N. 2023. Digital public health interventions at scale: the impact of social media advertising on beliefs and outcomes related to COVID vaccines. Proc Natl Acad Sci U S A. 120:e2208110120. 36701366 10.1073/pnas.2208110120 PMC9945974

[36] Batteux E, Mills F, Jones LF, Symons C, Weston D. 2022. The effectiveness of interventions for increasing COVID-19 vaccine uptake: a systematic review. Vaccines (Basel). 10:386. 35335020 10.3390/vaccines10030386 PMC8949230

[37] McManus J, Constable M, Bunten A, Chadborn T. 2018. Improving people's health: applying behavioural and 7 social sciences to improve population health and wellbeing in England. [accessed 2024 March 28]. https://assets.publishing.service.gov.uk/media/5bb21dd2e5274a3e0d7af9e0/Improving_Peoples_Health_Behavioural_Strategy.pdf.

[38] Ruggeri K, et al 2023. A synthesis of evidence for policy from behavioural science during COVID-19. Nature. 625:134–147. 38093007 10.1038/s41586-023-06840-9 PMC10764287

[39] Voelkel JG, et al 2022. Megastudy identifying effective interventions to strengthen Americans’ democratic attitudes, *Strengthening Democracy Challenge*. [accessed 2024 Mar 28]. https://www.strengtheningdemocracychallenge.org/paper.

[40] Vlasceanu M, Doell K, Bak-Coleman J, Bavel JJV. 19 November 2023. Addressing Climate Change with Behavioral Science: A Global Intervention Tournament in 63 Countries. PsyArXiv cr5at. 10.31234/osf.io/cr5at, preprint: not peer reviewed. PMC10849597 38324680

[41] Milkman KL, et al 2021. Megastudies improve the impact of applied behavioural science. Nature. 600:478–483. 34880497 10.1038/s41586-021-04128-4 PMC8822539

[42] Duckworth AL, Milkman KL. 2022. A guide to megastudies. PNAS Nexus. 1:pgac214. 36712333 10.1093/pnasnexus/pgac214 PMC9802435

[43] Egami N, Hartman E. 2023. Elements of external validity: framework, design, and analysis. Am Polit Sci Rev. 117:1070–1088.

[44] Druckman JN . 2022. Experimental thinking: a primer on social science experiments. Cambridge: Cambridge University Press.

[45] Chater N, Loewenstein G. 2022. The i-frame and the S-frame: how focusing on individual-level solutions has led behavioral public policy astray. Behav Brain Sci. 46:e147. 36059098 10.1017/S0140525X22002023

[46] Hagmann D, Ho EH, Loewenstein G. 2019. Nudging out support for a carbon tax. Nat Clim Change. 9:484–489.

[47] Sheeran P . 2002. Intention—behavior relations: a conceptual and empirical review. Eur Rev Soc Psychol. 12:1–36.

[48] Webb TL, Sheeran P. 2006. Does changing behavioral intentions engender behavior change? A meta-analysis of the experimental evidence. Psychol Bull. 132:249–268. 16536643 10.1037/0033-2909.132.2.249

[49] Rhodes RE, Dickau L. 2012. Experimental evidence for the intention–behavior relationship in the physical activity domain: a meta-analysis. Health Psychol. 31:724–727. 22390739 10.1037/a0027290

[50] Moehring A, et al 2023. Providing normative information increases intentions to accept a COVID-19 vaccine. Nat Commun. 14:126. 36624092 10.1038/s41467-022-35052-4 PMC9828376

[51] Lakens D . 2013. Calculating and reporting effect sizes to facilitate cumulative science: a practical primer for t-tests and ANOVAs. Front Psychol. 4:863. 24324449 10.3389/fpsyg.2013.00863 PMC3840331

[52] Coppock A . 2022. Persuasion in parallel. Chicago: University of Chicago Press.

[53] Coppock A, Hill SJ, Vavreck L. 2020. The small effects of political advertising are small regardless of context, message, sender, or receiver: evidence from 59 real-time randomized experiments. Sci Adv. 6:eabc4046. 32917601 10.1126/sciadv.abc4046 PMC7467695

[54] Tappin BM, Wittenberg C, Hewitt LB, Berinsky AJ, Rand DG. 2023. Quantifying the potential persuasive returns to political microtargeting. Proc Natl Acad Sci U S A. 120:e2216261120. 37307486 10.1073/pnas.2216261120 PMC10288628

[55] Viechtbauer W . 2010. Conducting meta-analyses in R with the metafor package. J Stat Softw. 36:1–48.

[56] Hewitt L, Tappin BM. 19 September 2022. Rank-heterogeneous effects of political messages: Evidence from randomized survey experiments testing 59 video treatments. PsyArXiv xk6t3. 10.31234/osf.io/xk6t3, preprint: not peer reviewed.

[57] Milkman KL, et al 2021. A megastudy of text-based nudges encouraging patients to get vaccinated at an upcoming doctor's appointment. Proc Natl Acad Sci U S A. 118:e2101165118. 33926993 10.1073/pnas.2101165118 PMC8157982

[58] Patel MS, et al 2023. A randomized trial of behavioral nudges delivered through text messages to increase influenza vaccination among patients with an upcoming primary care visit. Am J Health Promot. 37:324–332. 36195982 10.1177/08901171221131021 PMC10798571

[59] Hainmueller J, Hangartner D, Yamamoto T. 2015. Validating vignette and conjoint survey experiments against real-world behavior. Proc Natl Acad Sci U S A. 112:2395–2400. 25646415 10.1073/pnas.1416587112 PMC4345583

[60] Coppock A, Green DP. 2015. Assessing the correspondence between experimental results obtained in the lab and field: a review of recent social science research. Polit Sci Res Methods. 3:113–131.

[61] Bartoš V, Bauer M, Cahlíková J, Chytilová J. 2022. Communicating doctors’ consensus persistently increases COVID-19 vaccinations. Nature. 606:542–549. 35650433 10.1038/s41586-022-04805-y PMC9200639

[62] Bokemper SE, Gerber AS, Omer SB, Huber GA. 2021. Persuading US white evangelicals to vaccinate for COVID-19: testing message effectiveness in fall 2020 and spring 2021. Proc Natl Acad Sci U S A. 118:e2114762118. 34845032 10.1073/pnas.2114762118 PMC8670490

[63] Green J, et al 2022. Using general messages to persuade on a politicized scientific issue. Br J Polit Sci. 53:698–706.

[64] Bokemper SE, Huber GA, James EK, Gerber AS, Omer SB. 2022. Testing persuasive messaging to encourage COVID-19 risk reduction. PLoS One. 17:e0264782. 35320285 10.1371/journal.pone.0264782 PMC8942219

[65] James EK, Bokemper SE, Gerber AS, Omer SB, Huber GA. 2021. Persuasive messaging to increase COVID-19 vaccine uptake intentions. Vaccine. 39:7158–7165. 34774363 10.1016/j.vaccine.2021.10.039 PMC8531257

[66] Kaufman J, et al 2023. Effect of persuasive messaging about COVID-19 vaccines for 5- to 11-year-old children on parent intention to vaccinate. J Paediatr Child Health. 59:686–693. 36807943 10.1111/jpc.16374

[67] Wittenberg C, Tappin BM, Berinsky AJ, Rand DG. 2021. The (minimal) persuasive advantage of political video over text. Proc Natl Acad Sci U S A. 118:e2114388118. 34782473 10.1073/pnas.2114388118 PMC8617416

[68] Milkman KL, et al 2024. Megastudy shows that reminders boost vaccination but adding free rides does not. Nature. 631:179–188. 38926578 10.1038/s41586-024-07591-x PMC11222156

