# Supplementary material for: Using survey experiment pretesting to support future pandemic response
Source: PNAS Nexus. 2024 Oct 17;3(11):pgae469. doi: 10.1093/pnasnexus/pgae469 (PMC11523628; doi:10.1093/pnasnexus/pgae469)
Supplement: pgae469_Supplementary_Data [file pgae469_supplementary_data.pdf]

Using survey experiment pre-testing to support future pandemic response

Ben M. Tappin   Luke B. Hewitt

Contents

1 Computing weighted-average expected benefit **2**

2 Expected benefit of pre-testing for campaigns with budgets other than $105,000 **5**

3 Importance of possessing accurate knowledge of the parameter values **7**

4 Reviewing evidence for parameter values **12**

4.1 Average effect size of messages 12

4.2 Variability in message effects 26

1 Computing weighted-average expected benefit

Given that we have uncertainty about which set of parameter values is correct, Figure S1 shows the implication of assuming different distributions of uncertainty over these set of parameter values (for a $105,000 campaign). In Figure S1a1, for example, we represent each set of parameter values as being equally likely by assigning each a probability of 1/3. We then compute a weighted-average expected benefit of pre-testing across the three sets of parameter values, using the assigned probabilities as the weights. The result shows that the bare-minimum testing regime is expected to increase campaign impact on average, netting at least one-thousand extra attitudes/beliefs influenced and vaccinations received (Figure S1b1).

We can also consider other uncertainty distributions to examine how robust the returns to pre-testing are against more pessimistic distributions. For example, if we assign greater probability to the pessimistic scenario (p = 0.5) than either the best-guess (p = 0.3) or optimistic (p = 0.2) scenarios, the weighted-average benefit is still greater than 0 (Figure S1b2). Even when we assume the pessimistic scenario is substantially more likely (p = 0.8) than either of the other two scenarios (p = 0.15 and p = 0.05, respectively), Figure S1a3, pre-testing remains cost-effective under the bare-minimum testing regime (Figure S1b3). In summary, these results suggest that, even under conservative assumptions, survey experiment pre-testing is plausibly cost-effective for public health campaigns with a budget of $105,000.

Finally, we consider the impact of incorporating uncertainty over the parameter values for campaigns with different budgets. Figure S2 shows the weighted-average benefit estimates for each campaign budget and reinforces the above results: for the larger campaigns, pre-testing is robustly cost-effective — even if one places a large amount of probability (p = 0.8) on the pessimistic set of parameter values. For the smaller campaign, pre-testing is robust against some pessimism, but is not cost-effective under stronger pessimism.

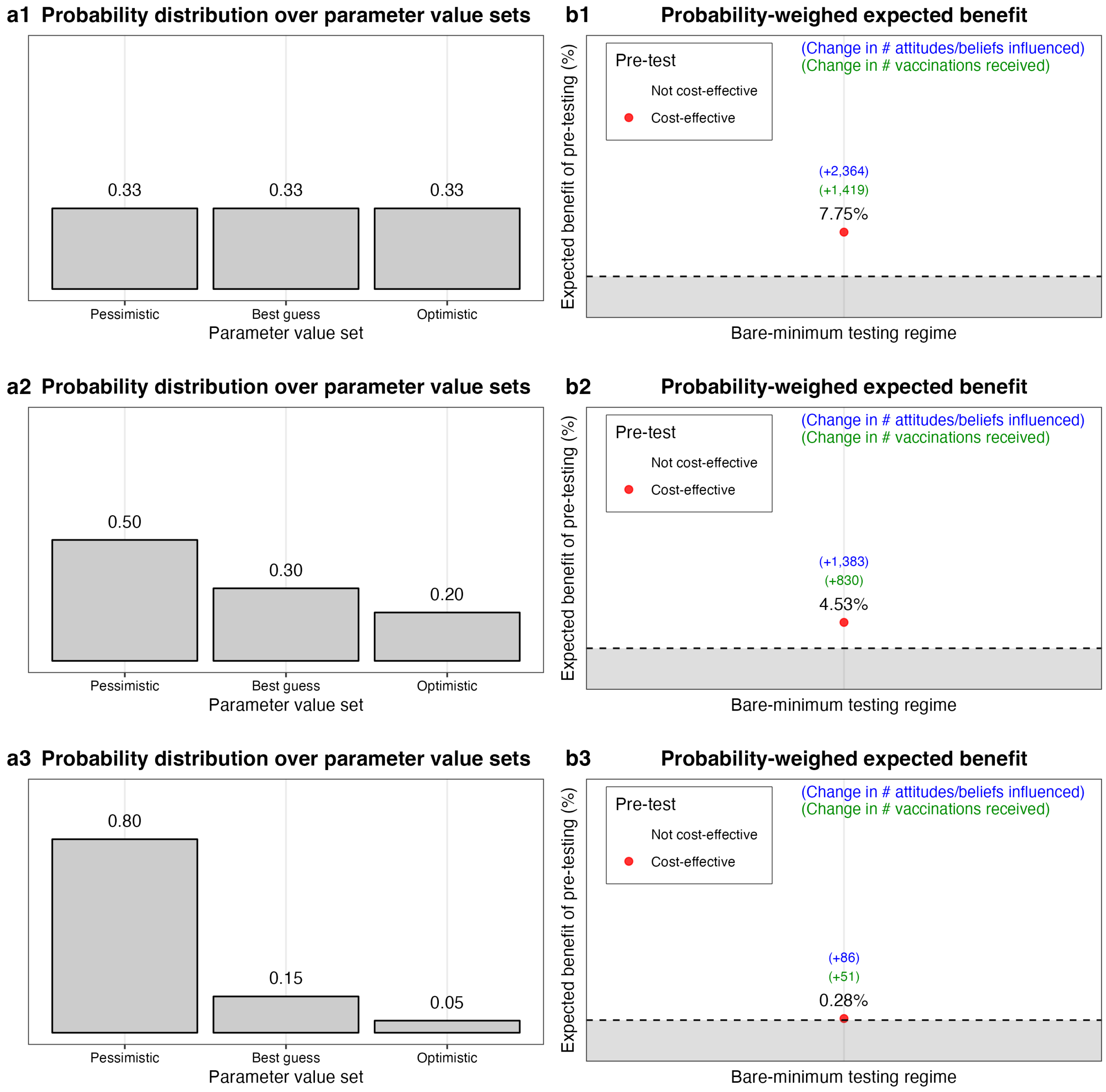

Figure S1: Weighted-average expected benefit of pre-testing for a campaign using the bare-minimum testing regime with a budget of $105,000.

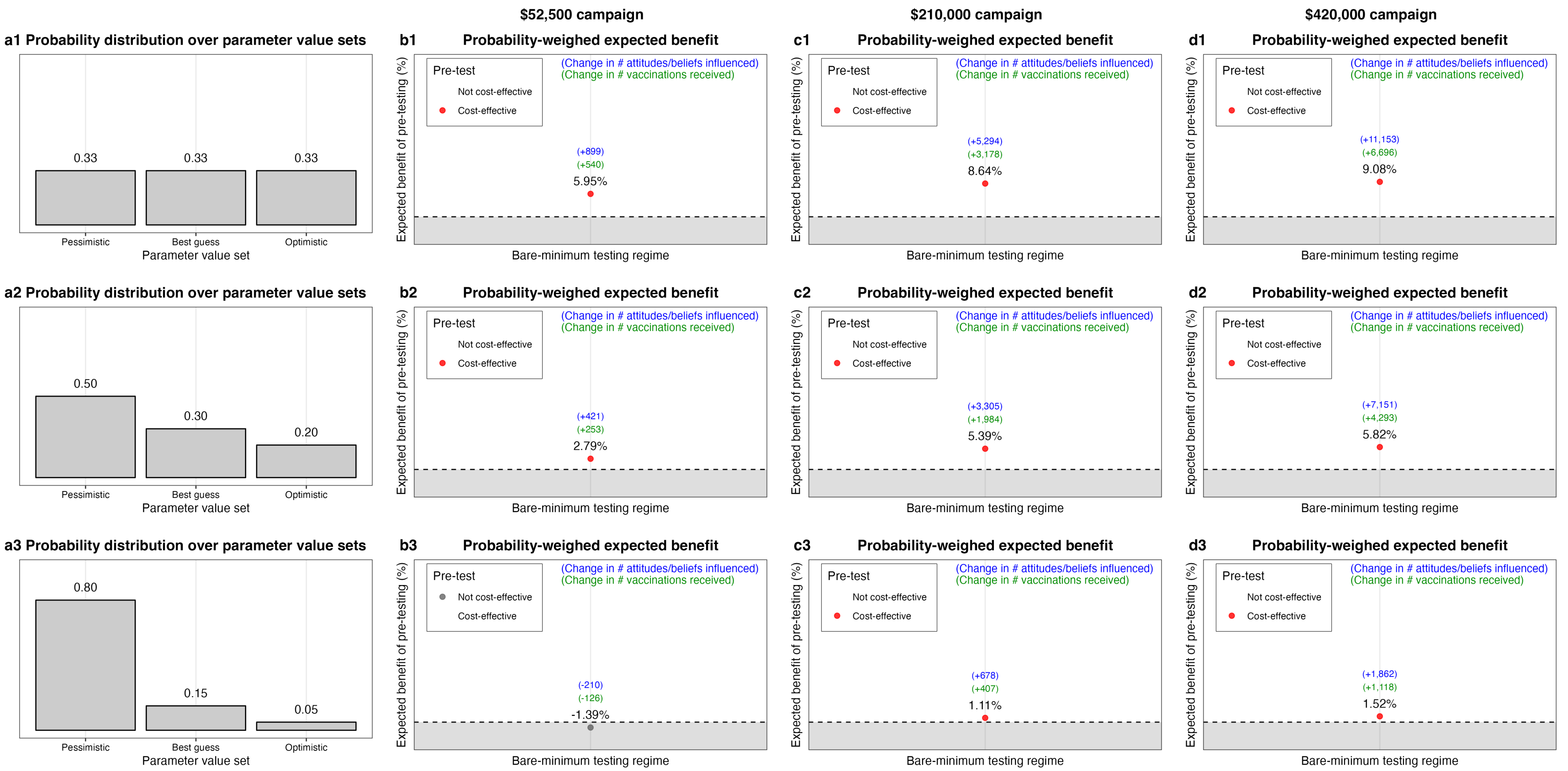

Figure S2: Weighted-average expected benefit of pre-testing for campaigns using the bare-minimum testing regime with different budgets.

2 Expected benefit of pre-testing for campaigns with budgets other than $105,000

Figures S3, S4 and S5 show, respectively, the estimated expected benefit of pre-testing for campaigns with budgets of $52,500, $210,000 and $420,000, with lower panels showing the corresponding parameter values (μ, $\frac{\mu}{\tau}$ and ρ).

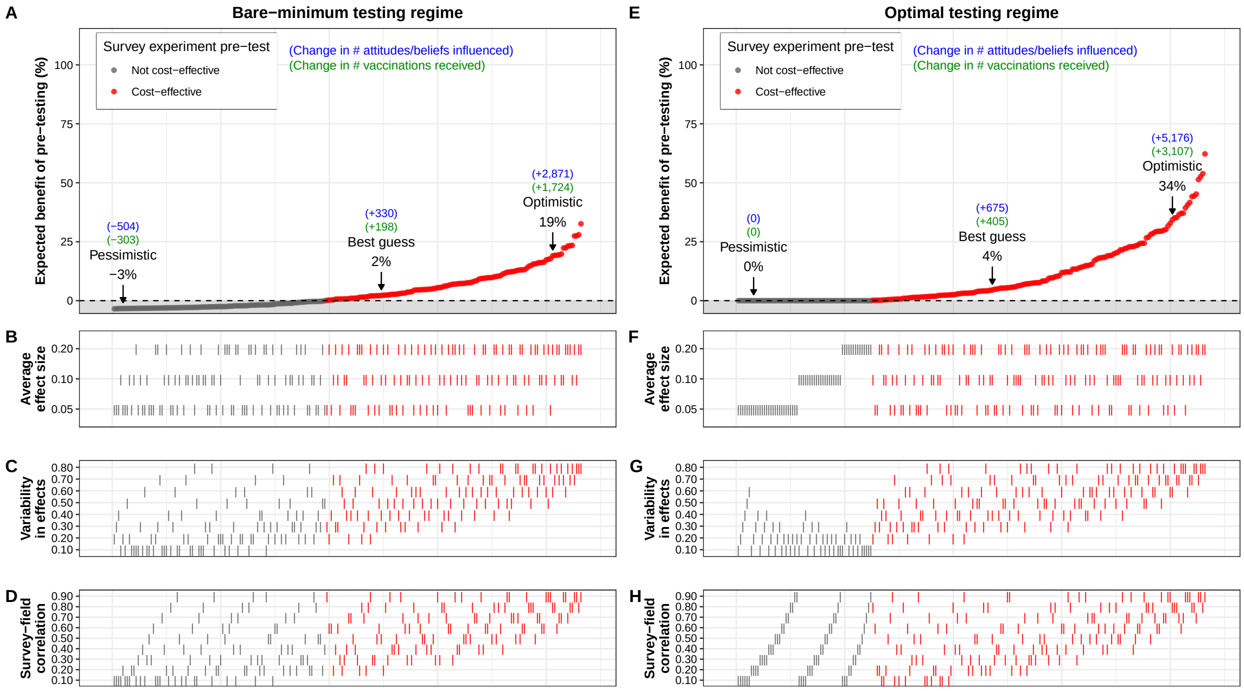

Figure S3: Estimated expected benefit of pre-testing for a campaign with a budget of $52,500.

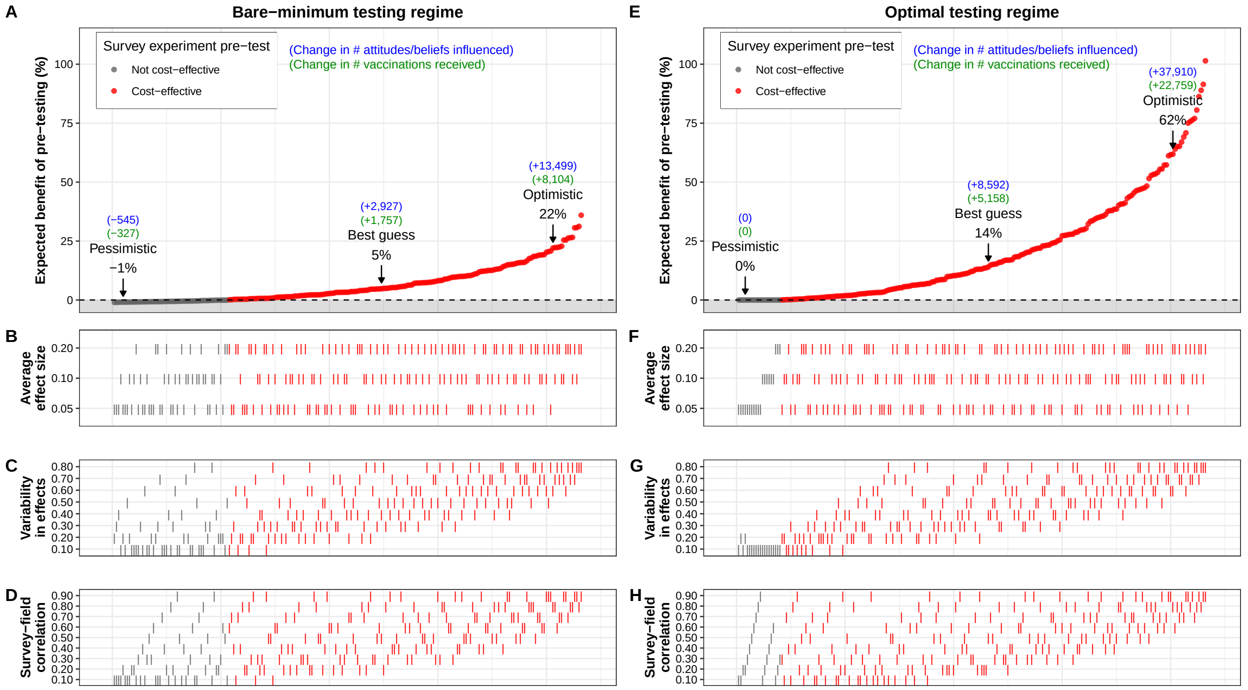

Figure S4: Estimated expected benefit of pre-testing for a campaign with a budget of $210,000.

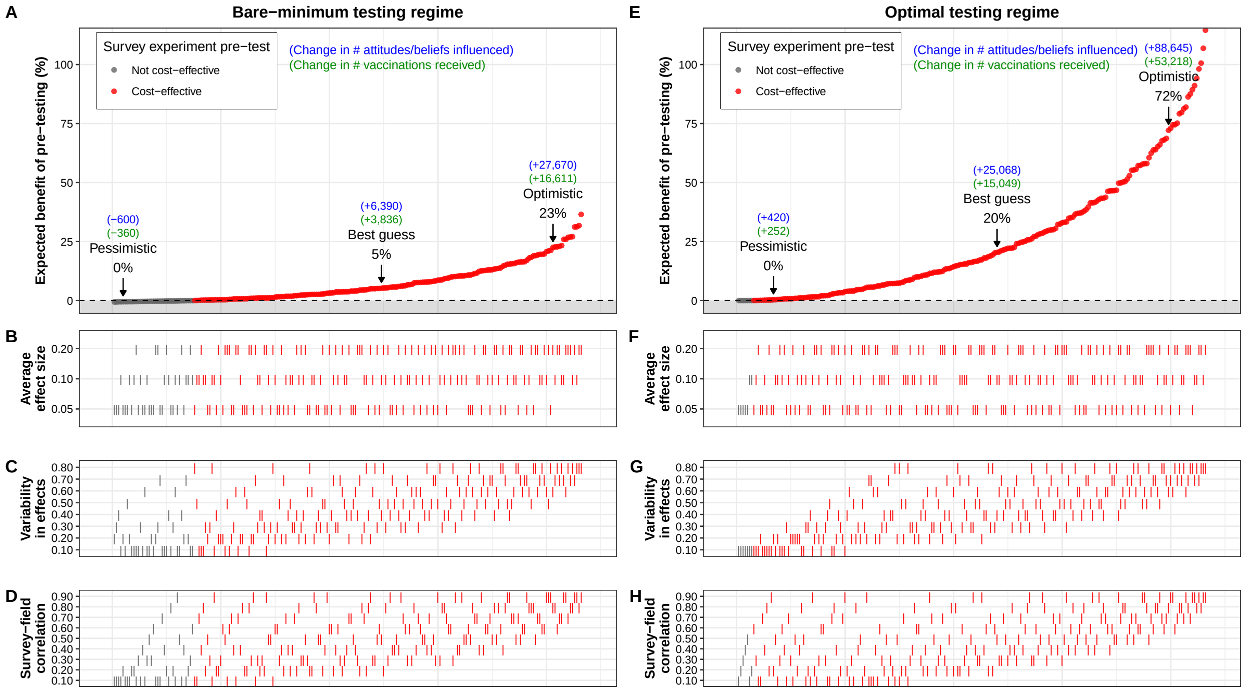

Figure S5: Estimated expected benefit of pre-testing for a campaign with a budget of $420,000.

3 Importance of possessing accurate knowledge of the parameter values

Figures S6, S7, S8 and S9 show, respectively, the estimated expected benefit of pre-testing for campaigns with budgets of \$52,500, \$105,000, \$210,000 and \$420,000 when the size of their pre-test is optimized for the best-guess set of parameter values, with lower panels showing the corresponding parameter values (μ, $\frac{\mu}{\tau}$ and ρ).

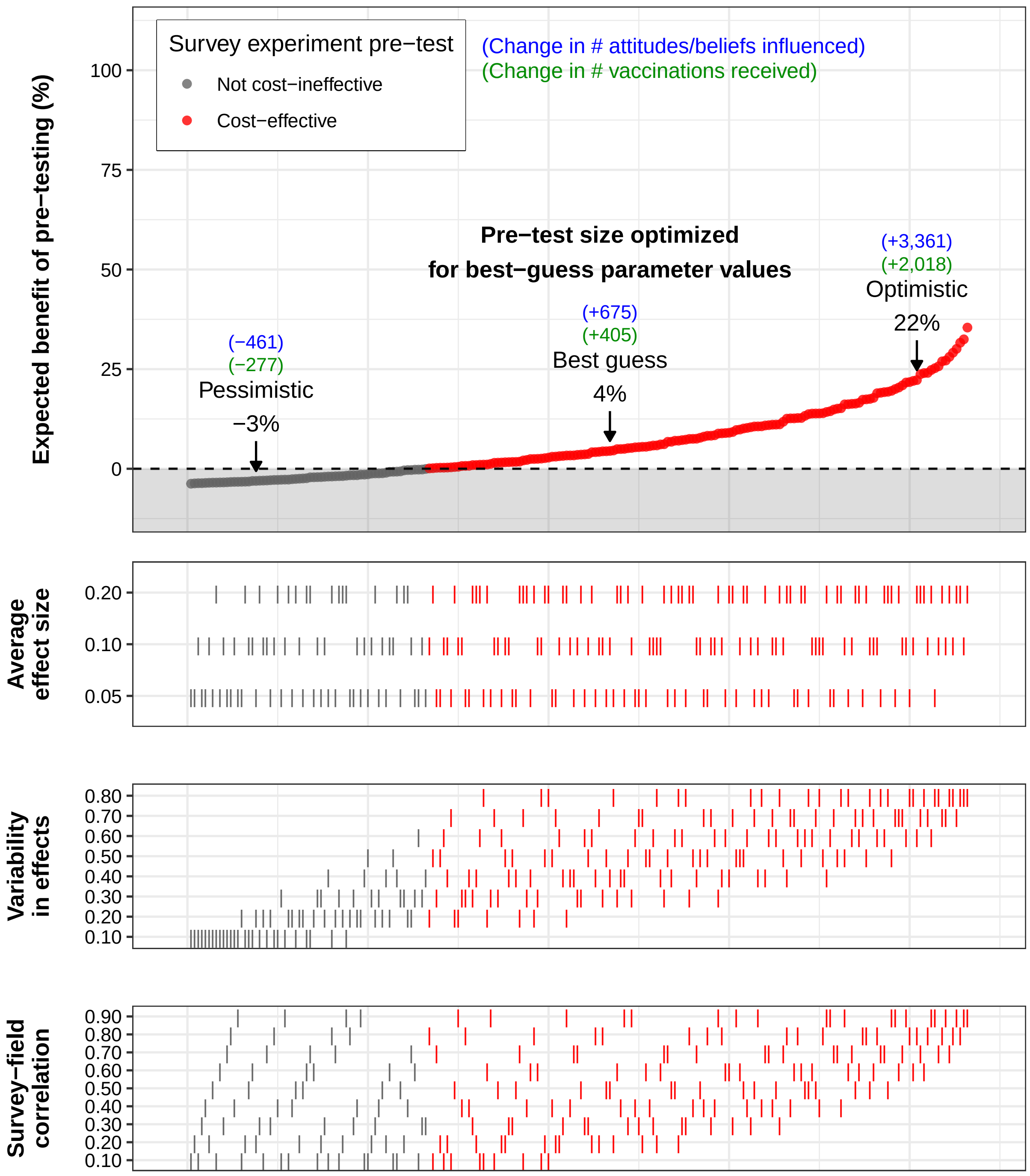

Figure S6: Estimated expected benefit of pre-testing for a $52,500 campaign when their pre-test regime is optimized for the best-guess set of parameter values.

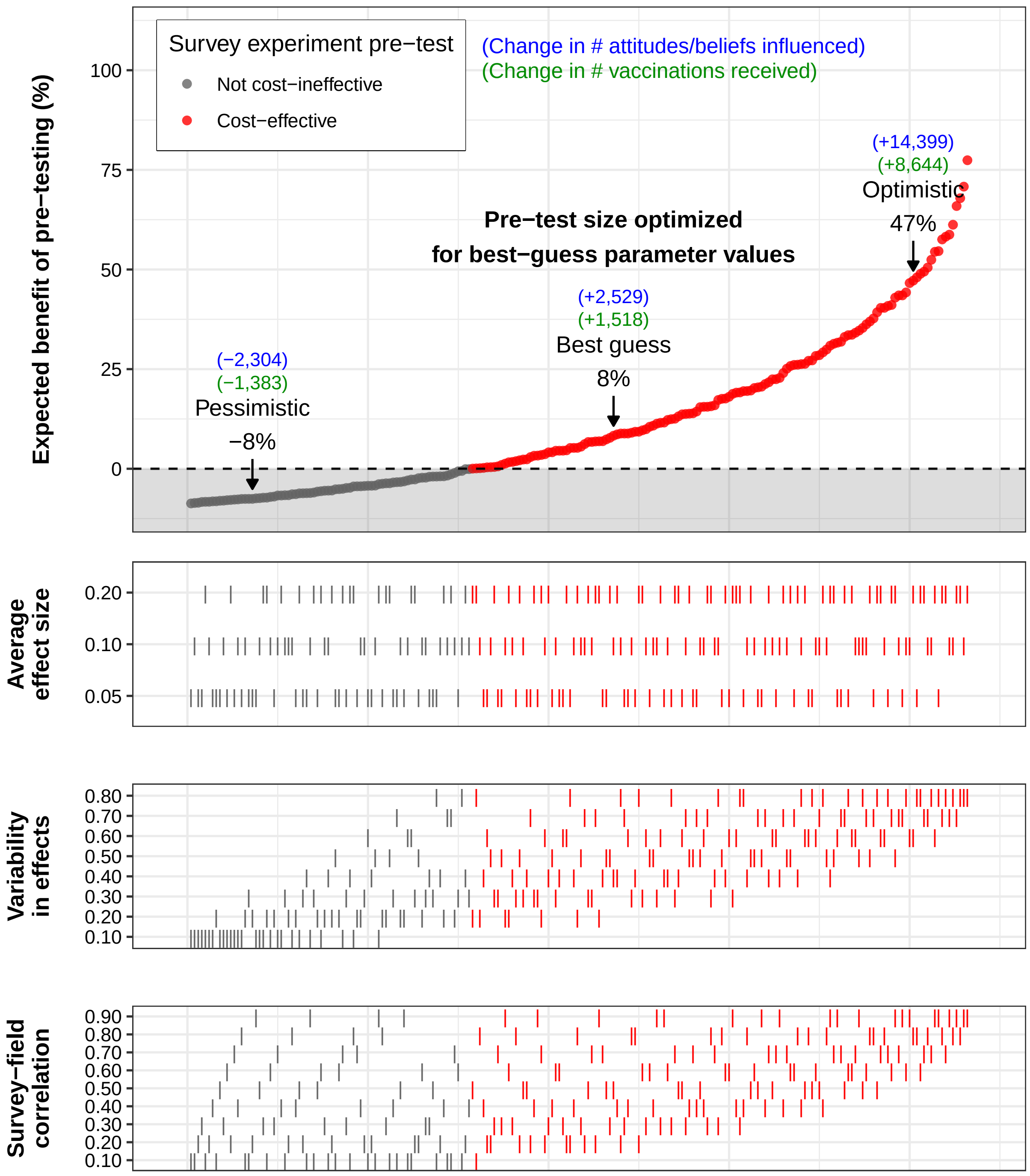

Figure S7: Estimated expected benefit of pre-testing for a $105,000 campaign when their pre-test regime is optimized for the best-guess set of parameter values.

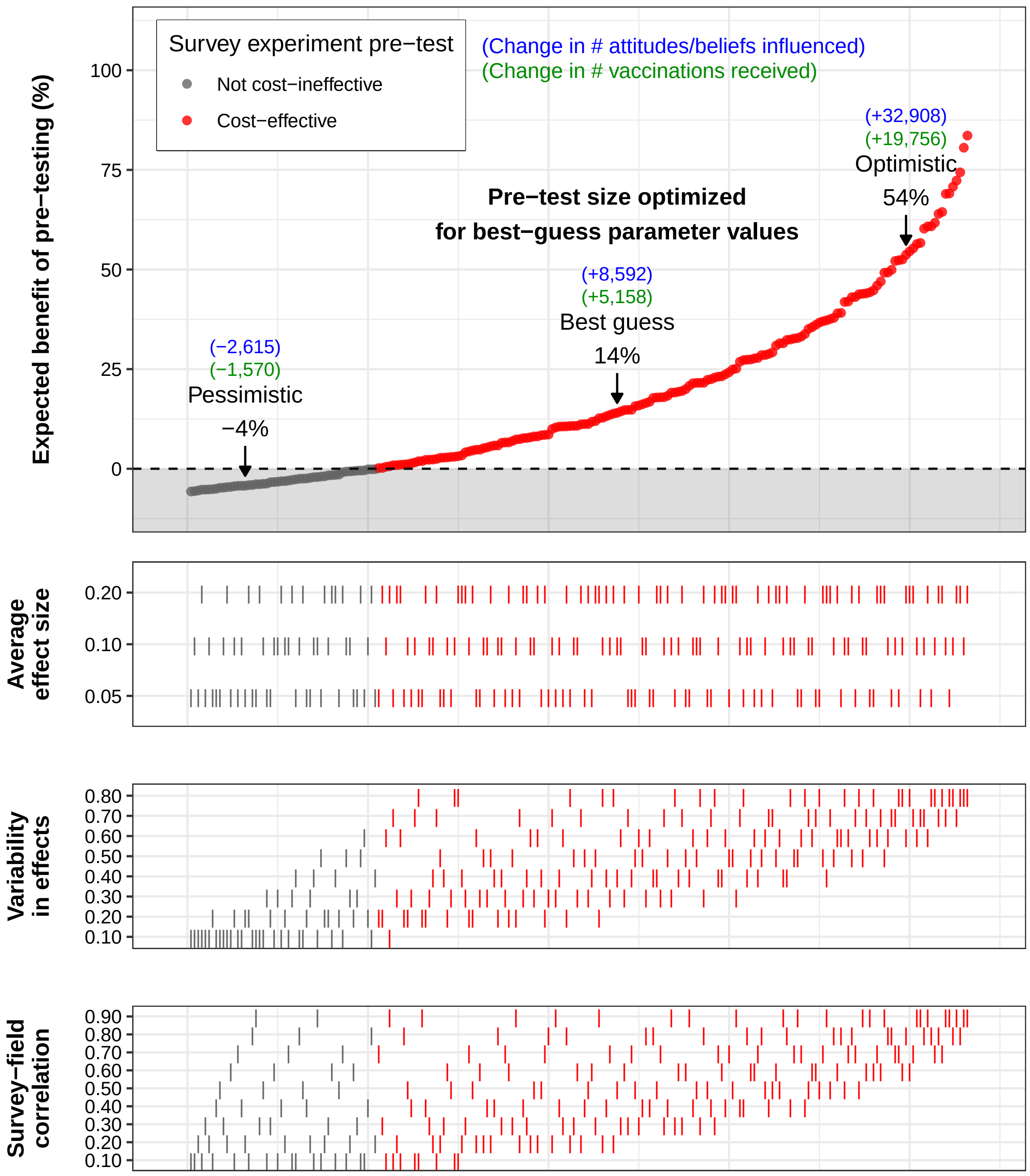

Figure S8: Estimated expected benefit of pre-testing for a $210,000 campaign when their pre-test regime is optimized for the best-guess set of parameter values.

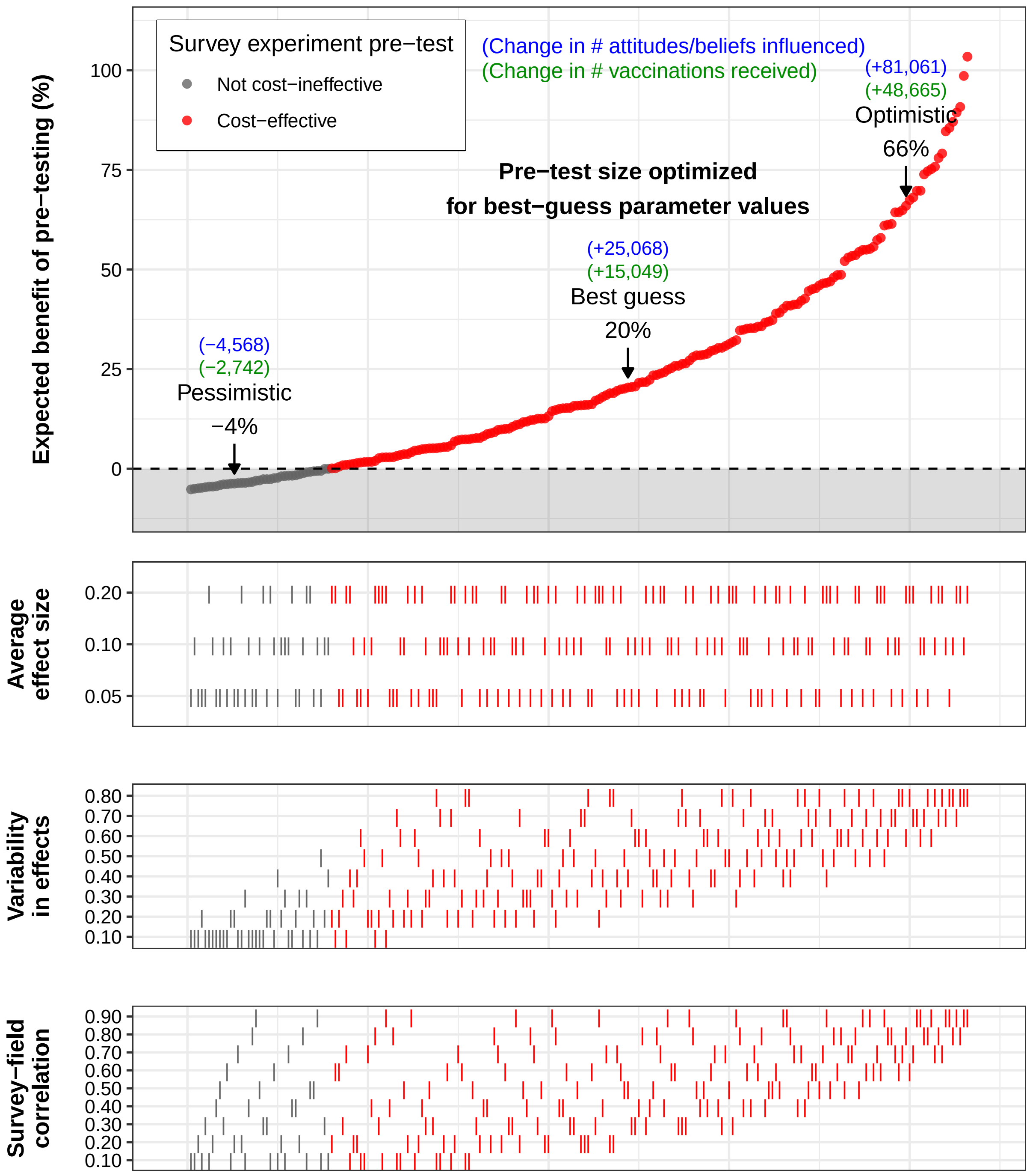

Figure S9: Estimated expected benefit of pre-testing for a $420,000 campaign when their pre-test regime is optimized for the best-guess set of parameter values.

4 Reviewing evidence for parameter values

Table S1 reports the studies we reviewed to estimate the average effect size of messages. Table S2 reports the studies we reviewed and re-analyzed to estimate the variability in message effects. For those studies that were sourced from the systematic review conducted by Batteux et al. (2022), we refer to that review for the full references.

4.1 Average effect size of messages

Table S1: Studies reviewed to estimate the average effect size of messages.

Author and study (where relevant)	Design	Sample size	# Treatments	Control group	ATE (in SDs)	Notes	Source
Batteux et al. 2021	Online RCT	328	2	No	0.21	An information treatment caused a greater decrease in vaccination intentions (standardized difference = 0.2) and perceived effectiveness (0.22) when it conflicted with a prior announcement that was certain vs. uncertain. We take the mean of these two values.	Batteux et al. 2022 systematic review
Behavioural Insights Team	Discrete choice experiment	4085	NA	No	NA	We were unable to determine the standardized ATE because there is no control group in the design of the experiment (discrete choice experiment).	Batteux et al. 2022 systematic review
Chen et al. 2021	Online RCT	413	8	No	NA	We were unable to determine the standardized ATE; there is no control group; supplement and data are not accessible.	Batteux et al. 2022 systematic review
Craig 2021	Discrete choice experiment	1153	NA	No	NA	We were unable to determine the standardized ATE because there is no control group in the design of the experiment (discrete choice experiment).	Batteux et al. 2022 systematic review

Table S1: Studies reviewed to estimate the average effect size of messages. *(continued)*

Author and study (where relevant)	Design	Sample size	# Treatments	Control group	ATE (in SDs)	Notes	Source
Dai et al. 2022	Online RCT	3181	1	Yes	0.16	There was one informational video intervention conducted in the survey RCT, and the outcome variable was intention to schedule an appointment and reported desire for the vaccine. They report Cohen's d for the video treatment as 0.16 (see Extended Data Table 6), averaging across all online RCTs and outcomes.	Batteux et al. 2022 systematic review
Davis et al. 2021	Online RCT	481	3	Yes	0.54	The covid-information-only treatment caused an increase in covid vaccination intentions of 0.39 SDs; the treatments that also contrasted this with the flu vaccine increased covid intentions by 0.68 SDs (estimates taken from the paper's abstract). We take the mean of these two values.	Batteux et al. 2022 systematic review
Duch et al. 2021	Online RCT	1628	3	Yes but not pure	NA	The outcome variable in this study is survey click-through-rate after viewing a video, which is a sufficiently different estimand to most of the other studies that we do not include it.	Batteux et al. 2022 systematic review

Table S1: Studies reviewed to estimate the average effect size of messages. *(continued)*

Author and study (where relevant)	Design	Sample size	# Treatments	Control group	ATE (in SDs)	Notes	Source
Freeman et al. 2021	Online RCT	15000	9	Yes but not pure	0.10	We compute the standardized ATE by first taking the reported condition marginal means, standard errors and sample sizes from the paper, and using these numbers to reverse-engineer the SD of the outcome variable. We then divide the reported ATE by this SD (thus standardizing it). The outcome variable is a vaccine hesitancy scale. Among the full sample the resulting standardized ATE is -0.02; among the strongly vaccine hesitant it is -0.18. We take the mean of these two values and convert to positive magnitude.	Batteux et al. 2022 systematic review
Han et al. 2021	Online RCT	1497	4	Yes	0.09	The treatments in this study weren't really directional in nature - that is, they weren't aimed at encouraging a particular behavior - rather they simply emphasized scientific uncertainty about COVID-19 in different ways. We thus should not expect them to have strong effects on the outcome variables. There were a variety of outcome variables examined. For the most relevant outcomes, "Intentions for COVID-19 Risk-Reducing Behaviors" and "Vaccination", reported standardized ATE magnitudes ranged from approximately zero to 0.18 (see Figure 2C and 2D and accompanying text). We thus take the midpoint of this range.	Batteux et al. 2022 systematic review

Table S1: Studies reviewed to estimate the average effect size of messages. *(continued)*

Author and study (where relevant)	Design	Sample size	# Treatments	Control group	ATE (in SDs)	Notes	Source
Kerr et al. 2021	Online RCT	4300	4	Yes	0.05	This paper reports two studies and investigates many different outcome variables. Most estimated effects are and statistically non-significant, but some treatment effects vs. control are statistically significant and in the 0.2 SD to 0.3 SD region (see Figures 1 and 4 and accompanying text). We err on the side of there being some nonzero but very small effect: 0.05.	Batteux et al. 2022 systematic review
McPhedran et al. 2021	Discrete choice experiment	2012	NA	No	NA	We were unable to determine the standardized ATE because there is no control group in the design of the experiment (discrete choice experiment).	Batteux et al. 2022 systematic review
Moehring et al. 2022	Online RCT	484000	1	Yes	0.03	This paper reports variations on a social norm treatment, but it is basically always a similar idea. They report an average effect of approximately 0.035 on a five point scale of vaccination intentions. The SD of a uniform distribution over 1-5 is approximately 1.4, so we compute the standardized ATE as 0.035/1.4 = 0.025.	Batteux et al. 2022 systematic review

Table S1: Studies reviewed to estimate the average effect size of messages. *(continued)*

Author and study (where relevant)	Design	Sample size	# Treatments	Control group	ATE (in SDs)	Notes	Source
Motta et al. 2021	Online RCT	7000	3	Yes	0.05	This paper estimates the effects of several different treatments, with effect sizes ranging from 0 to approximately 5pp. We thus take the midpoint of this range (2.5pp) as the average effect. The SD of a uniform 0-1 binary distribution is approximately 0.5 so we compute the standardized ATE as 0.025/0.5 = 0.05.	Batteux et al. 2022 systematic review
Palm et al. 2021	Online RCT	1123	6	Yes	0.20	In this paper there are two positive-focused treatment conditions, dubbed Safe and Effective and Willing, which had estimated effects of 0.36 and 0.43 respectively on a 1 to 7 scale. The SD of a uniform 1-7 distribution is approximately 2, so we compute the standardized ATEs as 0.36/2 = 0.18 and 0.43/2 = 0.22 respectively and then take the mean of these two values.	Batteux et al. 2022 systematic review
Pink et al. 2021	Online RCT	1480	2	Yes	0.03	This paper exposed US Republicans to in- or out-party cues or a control condition. Estimated effects of in-party vs. control condition ranged from approximately zero to 2.5pp across outcome variables. We take the midpoint of this range (1.25pp) as the effect size. The SD of a uniform 0-1 binary distribution is approximately 0.5 so we calculate the standardized ATE as 0.0125/0.5 = 0.025.	Batteux et al. 2022 systematic review

Table S1: Studies reviewed to estimate the average effect size of messages. *(continued)*

Author and study (where relevant)	Design	Sample size	# Treatments	Control group	ATE (in SDs)	Notes	Source
Serra-Garcia & Szech 2023	Online RCT	2100	Gradations	Yes	0.16	This paper explored financial incentives and opt-in vs. opt-out schemes on COVID-19 vaccination intention and demand for tests. For the financial incentive treatment, the randomization is graded i.e. increasing through dollar amounts. The effect for financial incentive is nonlinear and thus difficult to interpret: small incentives caused a decrease in intention/demand, but larger incentives caused an increase. The effects for the opt-out (vs. opt-in) condition ranged from 4pp to 12pp across different specifications for the intention/demand outcomes (see Table 1). We thus take the midpoint of these values (8pp) as the average effect. The SD of a uniform 0-1 binary distribution is approximately 0.5, so we compute a standardized ATE of 0.08/0.5 = 0.16.	Batteux et al. 2022 systematic review

Table S1: Studies reviewed to estimate the average effect size of messages. *(continued)*

Author and study (where relevant)	Design	Sample size	# Treatments	Control group	ATE (in SDs)	Notes	Source
Sinclair et al. 2023	Online RCT	661	5	Yes	0.06	This paper examined vaccination intentions and scores on a vaccine hesitancy scale. On the intentions outcome, the ATEs ranged from -0.04 to +0.34 points on a five point Likert scale - we take the midpoint (0.15) as the average effect - with an SD of approximately 1.25 (see Table 1). Thus, we compute a standardized ATE of 0.15/1.25 = 0.12. The effects on the vaccine hesitancy scale are approximately zero in the aggregate, so we halve the overall standardized ATE to 0.06.	Batteux et al. 2022 systematic review
Sprengholz & Betsch 2020	Online RCT	576	1	Yes	0.41	This paper reports that "participants in the herd immunity communication condition reported a mean likeliness to get vaccinated of 16.14 or 79.9% (SD = 4.67 or 24.6 percentage points), compared to 13.92 or 68.0% (SD = 6.25 or 32.9 percentage points) for those who received no information about herd immunity." This gives an ATE of 2.2 scale points, with an average SD of 5.46; equating to a standardized ATE of 2.2/5.46 = 0.41. Note that the disease was fictitious.	Batteux et al. 2022 systematic review

Table S1: Studies reviewed to estimate the average effect size of messages. *(continued)*

Author and study (where relevant)	Design	Sample size	# Treatments	Control group	ATE (in SDs)	Notes	Source
Sprengholz et al. 2021	Online RCT	2400	3	Unclear	NA	The outcome in this study is reactance (negative psychological reaction) towards vaccination and the treatments are not aiming to reduce it. It is also difficult to discern standardized effect sizes because the authors report primarily on interactions and the supplement doesn't make it clear whether the reported simple effects are standardized or unstandardized. We omit this study.	Batteux et al. 2022 systematic review
Sprengholz et al. 2022	Online RCT	782	1	Yes	0.07	This paper examined a legal incentive vs. no incentive condition to get vaccinated, for zero financial compensation. The point estimate on willingness to get vaccinated was 3.7pp higher on average in the legal incentive condition. Given the SD of a uniform 0-1 binary distribution is 0.5, this implies a standardized ATE of 0.037/0.5 = 0.074.	Batteux et al. 2022 systematic review
Strickland et al. 2021	Online RCT	1366	Various	Unclear	NA	Four experiments. It is difficult to discern the effect sizes of message exposure because the analysis is primarily an AUC analysis. We omit this study.	Batteux et al. 2022 systematic review

Table S1: Studies reviewed to estimate the average effect size of messages. *(continued)*

Author and study (where relevant)	Design	Sample size	# Treatments	Control group	ATE (in SDs)	Notes	Source
Taber et al. 2021	Online RCT	850	Various	No	NA	This paper reports two experiments: one on lottery structure and the other on framing loss/gain. The treatments are continuous through e.g. lottery structure. Not easy to discern relevant standardized ATEs. We omit this study.	Batteux et al. 2022 systematic review
Thorpe et al. 2022	Online RCT	361	2	Yes	0.00	This paper studied 4 outcome variables and 2 different treatments, giving 8 treatment effects. The corresponding estimated ATEs are all null effects whose point estimates bounce around all over the place (see Table 2). We code this as a treatment effect of zero overall.	Batteux et al. 2022 systematic review
Trueblood et al. 2022	Online RCT	1000	3	Yes	0.02	This paper examines three treatments where the outcome is how long people would wait for the vaccine. We take the mean of the three treatment effects (0.4155, -0.2133, -0.0427), which are measured on an 11-point outcome scale, and divide this mean by the approximate SD of a uniform distribution over 1-11 (i.e. 3.14). Thus giving an overall standardized ATE of 0.02.	Batteux et al. 2022 systematic review

Table S1: Studies reviewed to estimate the average effect size of messages. *(continued)*

Author and study (where relevant)	Design	Sample size	# Treatments	Control group	ATE (in SDs)	Notes	Source
Witus & Larson 2022	Online RCT	1632	3	Yes	0.15	This paper examines vaccination intentions and three treatments. We take the mean of the predicted probabilities of each treatment effect on "definitely" getting the vaccine (0.11, 0.075, 0.04; see Figure 1) and divide this mean by the SD of a uniform distribution over a binary 0-1 scale (i.e. 0.5). Thus, we compute the standardized ATE as 0.15.	Batteux et al. 2022 systematic review
Bartos et al. 2022	Online RCT	2000	1	Yes	0.09	This paper reports a longitudinal experiment. The treatment informs people of the consensus among doctors regarding the COVID-19 vaccines. Estimated treatment effects on beliefs and self-reported vaccination status range from 3pp to 6pp. We take the midpoint of this range (4.5pp) and divide it by the SD of a uniform distribution over a binary 0-1 variable (0.5); resulting in a standardized ATE of 0.045/0.5 = 0.09.	Snowball sampling / knowledge of literature
Wittenberg et al. 2021	Online RCT	3343	24	Yes	0.25	This paper studies dozens of treatment videos targeting COVID-19 beliefs, attitudes and behavioral intentions. The outcome variable is unique to each video. The overall average standardized ATE is reported as 0.25.	Snowball sampling / knowledge of literature

Table S1: Studies reviewed to estimate the average effect size of messages. *(continued)*

Author and study (where relevant)	Design	Sample size	# Treatments	Control group	ATE (in SDs)	Notes	Source
Shen et al. 2015	Lab and field RCTs	9330	25	Yes but not pure	0.13	This paper reports a meta-analysis of 25 studies, all of which are related to public health communication but not COVID-19 specifically. They include studies that compare the effect of narrative information against a control group that receives non-narrative statistical or factual information. The authors report effect size r, which we convert to Cohen's d here: https://www.escal.site/	Snowball sampling / knowledge of literature
Jordan et al. 2021	Online RCT	988	3	Yes	0.23	This paper reports several studies, however only study 1 contains a control group. Study 1 reports standardized ATEs of 0.17, 0.20 and 0.33. We take the mean of these values. Outcomes are COVID-19 related.	Snowball sampling / knowledge of literature
Kaufman et al. 2022	Online RCT	463	4	Yes	0.05	This paper examines treatments to encourage parents to covid-19 vaccinate their children. The primary outcome variable is probability the respondent answers "Definitely or probably will get a COVID-19 vaccine for child", coded 1 if so and 0 otherwise. Treatment effects are (in pp): -3.9, -0.8, 6.9, 7.8 (see Table 3). We take the mean of these values and divide by the SD of a uniform distribution over binary 0-1 (i.e. 0.5); giving a standardized ATE of 0.025/0.5 = 0.05.	Snowball sampling / knowledge of literature

Table S1: Studies reviewed to estimate the average effect size of messages. *(continued)*

Author and study (where relevant)	Design	Sample size	# Treatments	Control group	ATE (in SDs)	Notes	Source
Green et al. 2022	Online RCT	24682	5	Yes	0.07	This paper reports a study investigating the effects of five treatments to reduce COVID-19 vaccine resistance. They report the following effect magnitudes of each treatment (in pp): 5, 5, 3, 3, 2. We take the mean of these values and then divide by a uniform distribution over binary 0-1 (i.e. 0.5); thus giving a standardized ATE of 0.072.	Snowball sampling / knowledge of literature
Bokemper et al. 2021 study 1	Online RCT	855	6	Yes	0.08	This study examines several different COVID-19 outcomes and six treatments. We take the mean of the six treatment effects across all outcome variables (see Supplementary Table on the OSF) and divide it by the SD of a uniform distribution over binary 0-1 (0.5) to get the overall standardized ATE.	Snowball sampling / knowledge of literature
Bokemper et al. 2021 study 2	Online RCT	2419	5	Yes	0.02	This study examines several different COVID-19 outcomes and five treatments. We take the mean of the five treatment effects across all outcome variables (see Supplementary Table on the OSF) and divide it by the SD of a uniform distribution over binary 0-1 (0.5) to get the overall standardized ATE.	Snowball sampling / knowledge of literature

Table S1: Studies reviewed to estimate the average effect size of messages. *(continued)*

Author and study (where relevant)	Design	Sample size	# Treatments	Control group	ATE (in SDs)	Notes	Source
Bokemper et al. 2022 study 1	Online RCT	2568	10	Yes but not pure	0.03	This study examined various COVID-19 outcomes and ten treatments. Note that the control group was not a pure control but baseline persuasive information. We take the mean across treatment effects for each outcome variable, then divide by the SD of that outcome variable (reported in appendix table S2) to get the standardized overall ATE for each outcome. We then compute the overall standardized ATE by taking the mean across the standardized ATE for each outcome variable.	Snowball sampling / knowledge of literature
Bokemper et al. 2022 study 2	Online RCT	6000	3	Yes	0.04	This study examined five COVID-19 outcome variables and three treatments. We take the mean of the three treatment effects across all outcome variables (see appendix table S5) and divide it by the SD of a uniform distribution over binary 0-1 (0.5) to get the overall standardized ATE.	Snowball sampling / knowledge of literature

Table S1: Studies reviewed to estimate the average effect size of messages. *(continued)*

Author and study (where relevant)	Design	Sample size	# Treatments	Control group	ATE (in SDs)	Notes	Source
James et al. 2021 study 1	Online RCT	4361	11	Yes	0.17	This study examined three COVID-19 outcomes and eleven treatments. We take the mean across treatment effects for each outcome variable, then divide by the SD of that outcome variable (reported in appendix table S1) to get the standardized overall ATE for each outcome. We then compute the overall standardized ATE by taking the mean across the standardized ATE for each outcome variable. Note that the vaccination intention outcome is the combined version.	Snowball sampling / knowledge of literature
James et al. 2021 study 2	Online RCT	5014	6	Yes	0.08	This study examined three COVID-19 outcome variables and six treatments. We take the mean of the six treatment effects across all outcome variables (see appendix table S2) and divide it by the SD of a uniform distribution over binary 0-1 (0.5) to get the overall standardized ATE.	Snowball sampling / knowledge of literature

4.2 Variability in message effects

Table S2: Studies reviewed and re-analyzed to estimate the variability in message effects.

Author	Design	Sample size	# Treatments	Control group	Scaled SD	Notes	Domain	Source
James et al. 2021 study 1	Online RCT	4361	11	Yes	0.30	Greg Huber (author) provided the data over email. We first estimated the treatment effects from the response-level data. We then used random effects meta-analysis to estimate mean and SD in treatment effects, and then scaled the SD by dividing by the mean. We computed the scaled SD for each of three outcomes in the paper and then averaged across the scaled SDs to compute the overall scaled SD.	health, covid	Snowball sampling / knowledge of literature
James et al. 2021 study 2	Online RCT	5014	6	Yes	0.20	Greg Huber (author) provided the data over email. We first estimated the treatment effects from the response-level data. We then used random effects meta-analysis to estimate mean and SD in treatment effects, and then scaled the SD by dividing by the mean. We computed the scaled SD for each of three outcomes in the paper and then averaged across the scaled SDs to compute the overall scaled SD.	health, covid	Snowball sampling / knowledge of literature
Freeman et al. 2021	Online RCT	15000	9	Yes but not pure	NA	Paper says contact author for data. No reply to email attempts.	health, covid	Batteux et al. 2022 systematic review

Table S2: Studies reviewed and re-analyzed to estimate the variability in message effects. *(continued)*

Author	Design	Sample size	# Treatments	Control group	Scaled SD	Notes	Domain	Source
Bokemper et al. 2022 study 1	Online RCT	2568	10	Yes but not pure	1.11	Data were publicly available from the Harvard dataverse. We first estimated the treatment effects from the response-level data. We then used random effects meta-analysis to estimate mean and SD in treatment effects, and then scaled the SD by dividing by the mean. We computed the scaled SD for each of four outcomes, as per the paper and appendix, and then averaged across the scaled SDs to compute the overall scaled SD.	health, covid	Snowball sampling / knowledge of literature
Green et al. 2022	Online RCT	24682	5	Yes	0.41	Jon Green (author) provided the data over email. We first estimated the treatment effects from the response-level data. We then used random effects meta-analysis to estimate mean and SD in treatment effects, and then scaled the SD by dividing by the mean. We computed the scaled SD for the primary seven-point outcome variable.	health, covid	Snowball sampling / knowledge of literature

Table S2: Studies reviewed and re-analyzed to estimate the variability in message effects. *(continued)*

Author	Design	Sample size	# Treatments	Control group	Scaled SD	Notes	Domain	Source
Bokemper et al. 2021 study 1	Online RCT	855	6	Yes	1.73	Data were publicly available from the Harvard dataverse. We first estimated the treatment effects from the response-level data. We then used random effects meta-analysis to estimate mean and SD in treatment effects, and then scaled the SD by dividing by the mean. We computed the scaled SD for each of three outcomes, as per the paper, and then averaged across the scaled SDs to compute the overall scaled SD.	health, covid	Snowball sampling / knowledge of literature
Bokemper et al. 2021 study 2	Online RCT	2419	5	Yes	0.00	Data were publicly available from the Harvard dataverse. We first estimated the treatment effects from the response-level data. We then used random effects meta-analysis to estimate mean and SD in treatment effects, and then scaled the SD by dividing by the mean. We computed the scaled SD for each of three outcomes, as per the paper, and then averaged across the scaled SDs to compute the overall scaled SD.	health, covid	Snowball sampling / knowledge of literature

Table S2: Studies reviewed and re-analyzed to estimate the variability in message effects. *(continued)*

Author	Design	Sample size	# Treatments	Control group	Scaled SD	Notes	Domain	Source
Sinclair et al. 2023	Online RCT	661	5	Yes	1.60	Samantha Sinclair (author) provided the data over email. We first estimated the treatment effects from the response-level data. We used random effects meta-analysis to estimate mean and SD in treatment effects, and then scaled the SD by dividing by the mean. We computed the scaled SD for each of three outcomes, as per the paper, and then averaged across the scaled SDs to compute the overall scaled SD.	health, covid	Batteux et al. 2022 systematic review
Palm et al. 2021	Online RCT	1123	6	Yes	NA	The treatments in this study point in different directions i.e. some of the messages are casting doubt on the vaccines while others are encouraging vaccination. Thus, there are fewer than 5 treatments in the same direction. Ineligible.	health, covid	Batteux et al. 2022 systematic review
Chen et al. 2021	Online RCT	413	8	No	NA	No control group, ineligible.	health, covid	Batteux et al. 2022 systematic review
Milkman et al. 2022	Field RCT	689693	22	Yes	0.24	Data were publicly available from the Open Science Framework. Data was aggregated to the condition-level, so we first used logistic regression with multiple trials to estimate ATEs in log-odds space and then used random effects meta-analysis to estimate the mean and SD in treatment effects. Finally we scaled the SD by dividing by the mean.	health, non-covid	Snowball sampling / knowledge of literature

Table S2: Studies reviewed and re-analyzed to estimate the variability in message effects. *(continued)*

Author	Design	Sample size	# Treatments	Control group	Scaled SD	Notes	Domain	Source
Milkman et al. 2023	Field RCT	3662548	8	Yes	0.16	Data were publicly available from the Open Science Framework. We first estimated the treatment effects from the response-level data. We then used random effects meta-analysis to estimate the mean and SD in treatment effects and then we scaled the SD by dividing by the mean. Note this paper was not publicly available at the time of analysis, but was shared with us by the BCFG team.	health, covid	Snowball sampling / knowledge of literature
Milkman et al. 2021	Field RCT	47306	19	Yes	0.17	Data were publicly available from the Open Science Framework. Data was aggregated to the condition-level, so we first used logistic regression with multiple trials to estimate ATEs in log-odds space and then used random effects meta-analysis to estimate the mean and SD in treatment effects. Finally we scaled the SD by dividing by the mean.	health, non-covid	Snowball sampling / knowledge of literature
Coppock et al. 2020	Online RCT	34000	49	Yes	2.23	This paper reports two outcome variables: candidate favorability and vote choice. Estimated ATEs are reported as 0.0492 and 0.0072 respectively. Estimated SD(ATE) is reported as 0.0682 and 0.0222 respectively. Thus, the scaled SDs are 1.39 and 3.08 respectively. We take the mean of these values to compute the overall scaled SD.	politics	Snowball sampling / knowledge of literature

Table S2: Studies reviewed and re-analyzed to estimate the variability in message effects. *(continued)*

Author	Design	Sample size	# Treatments	Control group	Scaled SD	Notes	Domain	Source
Hewitt et al. 2023	Online RCT	500000	617	Yes	0.52	This paper reports two outcome variables, candidate favorability and vote choice, across three electoral contexts: 2018 downballot, 2020 downballot and 2020 presidential. As per the paper, 0.52 is reported as the average scaled SD across outcomes and contexts.	politics	Snowball sampling / knowledge of literature
Hewitt & Tappin 2022	Online RCT	40000	59	Yes	0.95	This papers reports on two policy issues and two broad sets of argument types (for vs. against each issue). The estimated SD in treatment effects and the mean treatment effect are reported, respectively, as follows (SD/mean, with implied scaled SD in parentheses): For-UBI: 0.06 / 0.11 (= 0.55); Against-UBI: 0.08 / 0.12 (= 0.67); For-USCA: 0.07 / 0.03 (= 2.33); Against-USCA: 0.03 / 0.12 (= 0.25). We take the mean of these four values to compute the overall scaled SD.	politics	Snowball sampling / knowledge of literature
